# Light‐Activation of DNA‐Methyltransferases

**DOI:** 10.1002/anie.202103945

**Published:** 2021-05-05

**Authors:** Jan Wolffgramm, Benjamin Buchmuller, Shubhendu Palei, Álvaro Muñoz‐López, Julian Kanne, Petra Janning, Michal R. Schweiger, Daniel Summerer

**Affiliations:** ^1^ Faculty of Chemistry and Chemical Biology TU Dortmund University Otto-Hahn Str. 4a 44227 Dortmund Germany; ^2^ Department of Epigenetics and Tumor Biology, Medical Faculty University of Cologne Kerpener Str. 62 50937 Köln Germany; ^3^ Max-Planck-Institute for Molecular Physiology Otto-Hahn-Str. 11 44227 Dortmund Germany

**Keywords:** DNA methyltransferases, epigenetics, genetic code expansion, optochemical biology

## Abstract

5‐Methylcytosine (5mC), the central epigenetic mark of mammalian DNA, plays fundamental roles in chromatin regulation. 5mC is written onto genomes by DNA methyltransferases (DNMT), and perturbation of this process is an early event in carcinogenesis. However, studying 5mC functions is limited by the inability to control individual DNMTs with spatiotemporal resolution in vivo. We report light‐control of DNMT catalysis by genetically encoding a photocaged cysteine as a catalytic residue. This enables translation of inactive DNMTs, their rapid activation by light‐decaging, and subsequent monitoring of de novo DNA methylation. We provide insights into how cancer‐related DNMT mutations alter de novo methylation in vivo, and demonstrate local and tuneable cytosine methylation by light‐controlled DNMTs fused to a programmable transcription activator‐like effector domain targeting pericentromeric satellite‐3 DNA. We further study early events of transcriptome alterations upon DNMT‐catalyzed cytosine methylation. Our study sets a basis to dissect the order and kinetics of diverse chromatin‐associated events triggered by normal and aberrant DNA methylation.

## Graphical abstract

We report light activation of DNA methyltransferases (DNMT) through the genetic encoding of a photocaged cysteine. This enables insights into the impact of cancer‐related DNMT mutations on in vivo catalysis, enables local and tuneable methylation via DNMT‐TAL‐effector fusions, and provides insights into methylation‐triggered transcriptome dynamics.

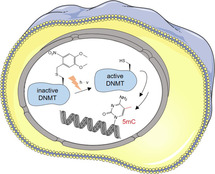

## Introduction

**Figure 1:**
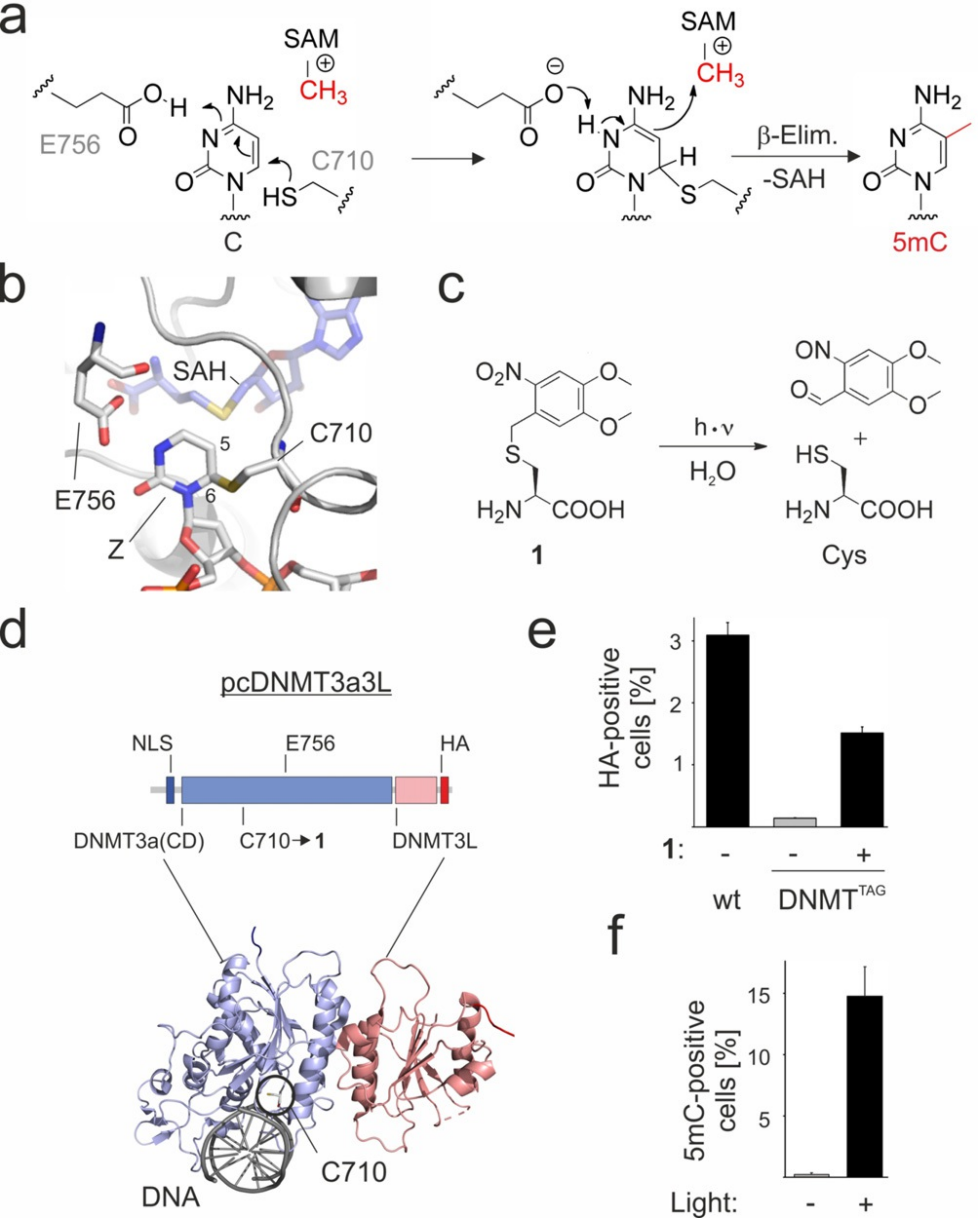
Light‐control of DNA methyltransferase catalysis. a) Mechanism of DNMT‐catalyzed cytosine methylation at C5. SAM: S‐adenosylmethionine; SAH: S‐adenosylhomocysteine. b) Crystal structure of human DNMT3a active site with catalytic C710 trapped with the cytosine analog zebularine (Z, pdb 6F57^[11]^). c) Structure and light‐deprotection of photocaged cysteine **1**. d) Domain and crystal structure (pdb 6F57^[11]^) overviews of pcDNMT3a3L with position of catalytic C710→**1** mutation for light‐activation. NLS: nuclear localization sequence. HA: hemagglutinin tag. e) Incorporation of **1** at pcDNMT3a3L amber codon (C710TAG) in HCT116 DKO cells analyzed by anti‐HA immunostaining and flow cytometry (FCM). Error bars show standard deviations from three independent biological replicates. f) Anti‐5mC immunostainings and FCM analysis of HCT116 DKO cells expressing pcDNMT3a3L 24 h after light or no light irradiation. Error bars show standard deviations from three independent biological replicates.

5‐Methylcytosine (5mC, Figure 1 a) is a dynamic regulatory element of mammalian genomes with key roles in transcription regulation, differentiation and development.^[1]^ 5mC is introduced and maintained by DNA‐methyltransferases (DNMT), and DNMT mutations are an early event in carcinogenesis.^[2]^ The ability to control DNMTs with spatiotemporal resolution in vivo would enable precise kinetic insights into how cancer‐related mutations alter DNMT function. Moreover, experimental control of 5mC levels at user‐defined genomic loci is of central interest in epigenome engineering, and spatiotemporal resolution would greatly enhance the precision of this approach.^[3]^

Compared to traditional small molecule effectors or transcriptional/translational control, light can offer direct control of protein functions with spatiotemporal resolution and unparalleled tunability, given that suited light‐responsive tools are available.^[4]^ In this direction, caged 5‐aza‐2′‐deoxycytidine (5Aza‐dC) derivatives have enabled light‐control of DNMT inhibition.^[5]^ However, 5Aza‐dC itself is not selective for specific cytosine‐directed DNMTs, and it acts by non‐specific incorporation into DNA and subsequent formation of DNMT‐DNA‐crosslinks, leading to DNA damage.^[6]^ Moreover, reduction of 5mC levels occurs via passive 5mC dilution, which limits temporal resolution. Alternatively, light‐activated recruitment of DNMT3a‐cryptochrome 2 protein (Cry2) fusions to specific target loci has been demonstrated via dimerization with the cytochrome‐interacting helix‐loop‐helix protein 1 (CIB1) fused to DNA binding domains.^[7]^ However, the use of such fusion constructs is restricted to methylation of specific loci, and it does not offer studying natural enzyme functions. Further, the mere recruitment of an overexpressed, constitutively active DNMT is expected to create a high methylation background.

We here report light‐control of DNMTs by directly masking an essential catalytic residue via substitution with a genetically encoded^[8]^ photocaged amino acid.^[9]^ This provides a general approach for the expression of transiently inactive wild type (wt) and mutant DNMT isoforms—as single enzymes and fusion constructs—followed by their activation with light. Using these tools, we study how cancer‐related mutations alter de novo methylation by a DNMT3a construct in vivo. We further demonstrate sequence‐specific and tuneable methylation of satellite‐3 (SATIII) DNA by light‐controlled, programmable DNMT‐transcription activator‐like effector (TALE) fusions, providing a strategy for advancing the control and precision of methylome engineering tools. Finally, we study early events of transcriptome reshaping by light‐activated DNMT3a methylation.

## Results and Discussion

DNMTs activate the cytosine nucleobase for attack of the S‐adenosylmethionine (SAM) cofactor methyl‐group via 1,4‐addition of a cysteine (C710) sulfhydryl group at the C6 atom followed by protonation of the N3 atom (Figure 1 a, b).^[10]^ To control the nucleophilicity of C710 in a light‐dependent manner, we targeted it for replacement with the noncanonical amino acid (ncAA) 4,5‐dimethoxy‐2‐nitrobenzyl‐L‐cysteine **1** (Figure 1 c).[^9b^, ^9c^] ncAA **1** can be incorporated into proteins in response to the amber codon (TAG) in yeast and mammalian cells by use of an *Escherichia coli* amber suppressor leucyl‐tRNA‐synthetase (LRS)/tRNA^Leu^ pair with engineered LRS aminoacylation and editing sites. Subsequent light‐irradiation of **1** leads to effective decaging to the natural cysteine residue (Figure 1 c).[^9b^, ^9c^]

We constructed a vector encoding the catalytic domain (CD, aa 612–912) of human DNMT3a (which is responsible for de novo methylation) fused to its activating interaction partner DNMT3L^[12]^ with a C‐terminal HA‐tag. The CD contains an amber codon at position C710 (Figure 1 d). For the expression of the LRS/tRNA^Leu^ pair, we employed a second vector that allows for amber suppression with **1** with high efficiency and fidelity (confirmed in a widely used mCherry‐GFP‐39TAG control; see Figures S1 and S4 for imaging and electrospray ionization (ESI)‐MS/MS characterization). We co‐transfected both vectors into HCT116 colon cancer cells that lack functional DNMT1 and DNMT3b genes (HCT116 DKO). These cells exhibit <5 % of the global 5mC level found in wild type HCT116 cells.^[13]^ After cultivation for 48 h in presence or absence of 0.05 mM **1**, we fixed and permeabilized the cells, and analyzed both populations by anti‐HA immunostaining and flow cytometry (FCM). We observed expression of the full‐length photocaged DNMT_C710→**1** protein construct (briefly: “pcDNMT3a3L”) only in presence of **1** (∼50 % compared to non‐amber DNMT), indicating high efficiency and fidelity of its incorporation at the target position (Figure 1 e, for characterization of amber suppression by a western blot, see Figure S5). To evaluate the light‐responsiveness of pcDNMT3a3L, we extended the FCM analyses to 5mC immunostainings (this assay provides sufficient sensitivity to measure the presence or absence of methylation by non‐amber wt or catalytically inactive mutant (E756A^[14]^) DNMT in the HCT116 DKO background; Figure S9). When we expressed pcDNMT3a3L for 48 h, removed **1** and irradiated the cells for 5 min with light (365 nm), we observed a 5mC signal only for the irradiated cell population (Figure 1 f and Figure S7). This shows that pcDNMT3a3L is indeed translated in an inactive state, and that it can be effectively activated with light.

DNMT3a is by far the most frequently mutated DNMT in haematological malignancies, but the effects of such mutations on its in vivo catalytic activity are still poorly understood.[^2^, ^15^] In vitro studies with synthetic DNAs as well as studies relying on mammalian expression of DNMT3a mutants and 5mC quantification have revealed a complex picture of cancer mutations, with both activity increases or decreases being possible.[^2^, ^11^] However, in vitro studies have limited relevance for enzyme functions under physiological conditions, and the expression of constitutively active mutants does not allow studying the kinetics of DNMT3a catalysis uncoupled from the kinetics of upstream processes, such as DNMT3a translation, folding, posttranslational modification and transport.

We employed pcDNMT3a3L as wt or bearing frequent cancer‐associated DNMT3a mutations in HCT116 DKO cells using light‐activation after 48 h. At this point, expression of wt and mutant DNMTs had reached a plateau of similar level (Figure S10), and **1** was removed from the medium to terminate the expression.

**Figure 2:**
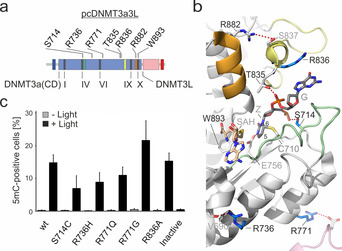
In vivo measurements of DNA methylation catalysis by pcDNMT3a3L with frequent cancer‐associated DNMT3a mutations using light‐control. a) Domain structure of pcDNMT3a3L with conserved DNMT motifs I–X and mutation positions analyzed in this study. Catalytic loop in green, TRD loop in yellow, part of homodimer interface in orange. b) Crystal structure of human DNMT3a active site bound to DNA (pdb 6F57^[11]^) with mutation positions analyzed by light activation shown as blue sticks. DNMT3L in pink, other color codes as in (a). c) Activity studies of pcDNMT3a3L cancer mutants conducted by anti‐5mC immunostainings and FCM. Measurements were conducted 24 h after light or no light irradiation. At this point, 5mC formation has not reached saturation for the wt (e.g., compare to R771G reaching even higher 5mC level). Error bars show standard deviations from three independent biological replicates.

We chose a range of mutations including several ones that had recently been studied in vitro in the specific context of DNMT3a3L.^[11]^ Though a variety of differences between in vitro and in vivo experimental conditions preclude a quantitative comparison of individual mutant activities, the similar DNMT constructs used in the in vitro study makes it the best available reference for comparisons between the two conditions. The mutations had different locations in this complex: DNMT3a and DNMT3L form a tetramer with two central DNMT3a molecules interacting via the homodimer interface, and two DNMT3L, each interacting with one DNMT3a via the opposite face.^[11]^ The DNMT3a catalytic domain harbors ten highly conserved motifs (I‐X, Figure 2 a) that are involved in SAM binding (I and X) and catalysis (e.g. IV and VI). DNA binding of DNMT3a is mainly mediated by the target recognition domain (TRD) loop (aa 831–848), the catalytic loop (aa 707–721, containing C710), and the homodimer interface (partly constituted by aa 881–887, Figure 2 a,b).^[11]^

The most frequently mutated residue of DNMT3a in cancers is R882, which is located at the homodimer interface, and undergoes a stabilizing interaction with TRD loop residue S837 (Figure 2 b).^[16]^ In preliminary studies, we found that the most prevalent mutants R882H and R882C cannot form 5mC to a detectable level when expressed in constitutively active form (Figure S9). We observed the same for mutations of T835 and W893 that interact with the CpG‐bridging phosphate and the SAM cofactor, respectively (Figure S9). We then conducted activity measurements with light activated DNMT via assessing 5mC formation 24 h after light irradiation. At this time, 5mC formation is still dynamic, offering relative activity comparisons between the mutants studied in our experiment (see Figure 2 c). We first studied the isosteric mutation S714C that is located in the catalytic loop and is expected to selectively delete a hydrogen bond to the CpG 5′‐phosphate by a defined O→S atomic substitution (Figure 2 b). This mutant exhibited ∼50% 5mC signal compared to wt pcDNMT3a3L after light activation, being in a similar range as observed for the DNMT3a CD in vitro but different to observations in murine ES cells, where this mutant is inactive (Figure 2 c).[^11^, ^17^, ^18^]

We next studied mutation R736H, involved in stabilizing the active site fold by a helix‐helix interaction (Figure 2 b). Though located at the interface to DNMT3L, R736H does not strongly affect the activation by DNMT3L in vitro.^[17]^ Conflicting results have been reported with DNMT3a CD for different substrates in vitro, ranging from 4‐fold increased to completely abolished activity.[^17^, ^18^] Our light activation measurements revealed a reduction in activity to ∼60 % (Figure 2 c). Another frequently mutated residue of the same interface is R771, being engaged in a salt bridge to DNMT3L (Figure 2 b). The two cancer mutations R771Q and R771G show markedly increased in vitro methylation (7‐ and 3‐fold higher than wt, respectively), and no or only slight reduction in DNMT3L activation.^[17]^ Surprisingly, we observed the opposite: R771Q slightly reduced, and R771G enhanced methylation 1.5‐fold compared to wt DNMT. Finally, we were interested in the mutation R836A located in the TRD loop that interacts with the target CpG‐guanine via a water‐mediated hydrogen bond (Figure 2 b; DNA interaction not shown).^[11]^ R836 is involved in cooperative DNA binding by DNMT3a multimers.^[19]^ We observed no alterations for the R836A mutation itself, being in agreement with in vitro and cellular studies (Figure 2 c).^[11]^ Overall, our light activation studies afforded in several cases surprisingly different activity data than studies conducted in vitro or with constitutively active mutants in other cell contexts would suggest. We thus expect that a future extension of our light activation technique to time‐resolved studies with such mutants and using full‐length DNMT3a will be instrumental to further dissect the exact nature of their malfunction.

**Figure 3:**
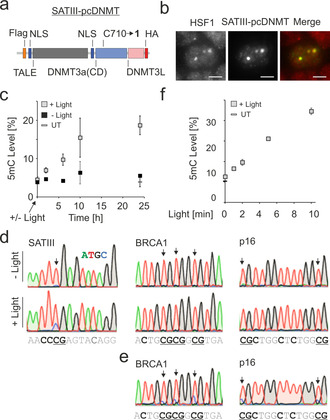
Light‐controlled, targeted methylation of SATIII loci. a) Domain structure of SATIII‐pcDNMT construct. b) Images of heat‐stressed HEK293T cells expressing SATIII‐pcDNMT immunostained with anti‐HA and anti‐HSF1 antibodies. Scale bar is 5 μm. c) Kinetics of SATIII methylation with SATIII‐pcDNMT after light irradiation and no light controls. 5mC was quantified by bisulfite PCR and Illumina amplicon deep sequencing. Error bars show standard deviations from three independent biological replicates. UT=untransfected + light. d) Methylation of SATIII and two off‐target loci by SATIII‐pcDNMT 24 h after irradiation analyzed by Sanger sequencing of bisulfite PCR products. gDNA sequence is shown below with Cs bold and CpG underlined. e) Methylation of the BRCA1 and p16 loci of (d) with TALE‐DNMT constructs designed to specifically target the BRCA1 and p16 loci, respectively. f) Light dosage dependence of SATIII methylation in experiments conducted as in (c). 5mC was quantified by bisulfite PCR and pyrosequencing. Error bars show standard deviations from three independent biological replicates. UT=untransfected.

The ability to selectively deposit 5mC in user‐defined genomic sequences is a key goal in epigenome engineering. This enables establishing causalities between local 5mC and various other chromatin features, including transcriptional activity.^[3]^ Compared to the current approach for light control of local methylation based on Cry2‐recruitment of constitutively active DNMT to a programmable DNA binding domain,^[7]^ a direct catalysis control of programmable DNMT constructs by light would enable locus‐specific DNA methylation with enhanced spatiotemporal control and lower propensity for background methylation. Moreover, the amount of locally deposited 5mC may be precisely tuned via the light dosage. We designed a construct of a transcription‐activator‐like effector (TALE) domain targeted to a sequence of SATIII pericentromeric DNA fused to pcDNMT3a3L (“SATIII‐pcDNMT”, Figure 3 a,^[20]^ Figure S20 shows vector map). SATIII DNA is the origin of nuclear stress bodies (nSB, a type of stress‐induced membrane‐less organelle^[21]^), and exhibits aberrant methylation in several cancers.^[22]^ We confirmed high efficiency and fidelity of amber suppression with **1** for this construct (Figures S2 and S3). We then evaluated SATIII‐pcDNMT for correct, selective genomic localization by expressing it in HEK293T cells and subjecting the cells to heat‐stress (1 h, 44 °C). We imaged the cells after conducting a co‐immunostaining for the HA‐tag of SATIII‐pcDNMT and for endogenous HSF1, a transcription factor recruited to SATIII upon heat stress. Co‐localization indicated correct target binding of SATIII‐pcDNMT (Figure 3 b and Figure S12).

We next irradiated the cells with light for 5 min and conducted time‐resolved 5mC quantification at a SATIII CpG by bisulfite PCR and Illumina amplicon deep sequencing (see SI for details). We observed a rapid increase of 5mC over the first 10 h, followed by a slower increase up to 24 h (Figure 3 c, see Figure S13 for additional controls).

This increase was strictly light‐dependent, indicated by the 5mC levels of the non‐irradiated samples that did not exceed the level of an untransfected control (Figure 3 c). Moreover, we observed a similar result for a second CpG in the amplicon (Figure S14).

To evaluate the locus‐specificity of methylation by SATIII‐pcDNMT, we conducted bisulfite PCRs of SATIII and two off‐target loci in the BRCA1 and p16 genes with DNA of cells harvested 24 h after light. Sanger sequencing revealed methylation of SATIII, but not BRCA1 or p16, showing locus‐specific methylation by SATIII‐pcDNMT (Figure 3 d and Figure S15). In turn, we could effectively methylate both off‐target loci with dedicated, BRCA1 and p16‐targeting TALE‐DNMT constructs, indicating that the absence of off‐target methylation by SATIII‐pcDNMT is due to high targeting selectivity, and not to generally inaccessible chromatin states at these loci (Figure 3 e; for an evaluation of the methylation window of a representative TALE‐DNMT construct, see Figure S16). Finally, we were interested, if local perturbation of 5mC by SATIII‐pcDNMT could be precisely tuned by applying different light dosages. Indeed, we observed a clear dependence of 5mC levels on the irradiation time up to 10 min (Figure 3 f; after this time, the majority of **1** is decaged in in vitro reference experiments, see Figure S17). This dosing ability may provide a convenient means for controlling the strength of 5mC‐triggered effects in local chromatin, such as the recruitment or release of 5mC‐responsive DNA binding factors or transcriptional activity of a locus.

**Figure 4:**
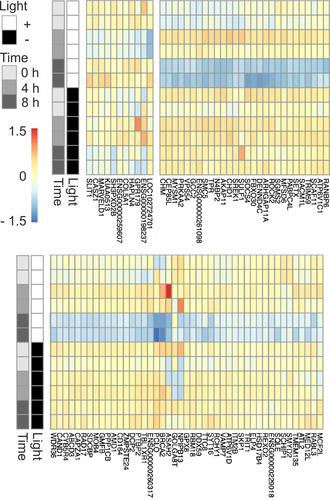
Light‐activated transcriptome regulation by pcDNMT3a in HEK293T cells. Gene expression patterns from mRNA‐Seq 0 h, 4 h and 8 h after light‐activation of pcDNMT3a. Two exemplary groups of differentially expressed genes with distinct expression patterns are shown (independent biological duplicates, full image in Figure S18) as log2‐fold change against average expression over all conditions; differential light‐dependent expression at a false discovery rate of <0.05.

5mC is an important regulator of chromatin condensation, with transcriptional downregulation as an ultimate biological consequence.^[1]^ To evaluate, if our light‐activated methylation would enable revealing early events in system‐wide transcriptional downregulation, we expressed wt pcDNMT3a (CD without DNMT3L) in HEK293T cells and activated it with light. We then measured mRNA expression levels on the transcriptome‐wide level by mRNA‐Seq at zero, four and eight hours after activation. We observed significant light‐dependent changes in transcription levels of a larger number of genes both after 4 and 8 h (Figure 4 shows representative examples; see Figure S18 and SI tables for full data analyses). The vast majority of genes thereby showed downregulation, albeit with different time profiles. A small group of fifteen genes was downregulated already after 4 h, including regulators involved in transcription activation, histone modification, and cell proliferation (e.g., CASZ1, SIK1, MARVELD1). Interestingly, the majority of these genes was again upregulated after 8 h (Figure 4, top left block). The largest group (73 genes) however showed a somewhat slower onset, with significant downregulation observed after 8 h. Strikingly, this group included a number of factors involved in central processes of signal transduction, chromatin regulation and RNA processing. The former included RABL3, DENND4C (a RAB10 activator^[23]^) and CHM (a subunit of the Rab GGTase complex^[24]^). Chromatin factors with direct, general roles in transcription were MED12L that is part of the mediator complex involved in activation of most RNA Pol II‐transcribed genes^[25]^ and SETX, a modulator of RNA Pol II interactions with chromatin.^[26]^ We also observed an early downregulation of ELP4, being part of a histone acetyltransferase complex associated with RNA Pol II.^[27]^ Other factors had general roles in chromatin regulation, such as CHD1, an ATP‐dependent chromatin remodeler, SMYD2, a methyltransferase of H3K4me3, Rb1 (involved in maintenance of heterochromatin via recruitment of e.g., SUV39H1), MYSM1, a deubiquitinase (DUB) of histone H2A, RCHY1, a ubiquitin ligase of HDAC1, and PRKAA2, a kinase targeting histone H2B and HDAC5.^[28]^ Strikingly, several of the factors are also direct modifiers of p53 (SMYD2, PRKAA2 and RCHY1). Factors involved in RNA binding, processing and transport were REXO2, RBM18, PTBP2, DCP2, RANBP6, SCAF11, PABPC4L, SREK1, and TPR (Figure 4).

These experiments show that downstream effects of de novo methylation can be studied for our light‐activatable DNMT on the system‐wide level. For the here employed model, we obtain insights into the initial target genes of pcDNMT3a and the early regulatory output of its methylation activity on the transcriptome level. The observed downregulation of a large number of genes with general, not gene‐specific regulatory functions suggests that the early DNA methylation events in this model have a higher‐order role in the regulation of downstream chromatin‐associated events.

## Conclusion

In conclusion, we report light‐control of DNMT catalysis in cells by replacing a catalytically essential cysteine residue with a genetically encoded photocaged cysteine. This enables the expression of individual, wild type or mutant DNMTs in a transiently inactive state followed by their rapid activation. Given their highly conserved mechanism, we anticipate that this strategy is transferrable to other DNMTs, as well. We reveal the impact of cancer‐associated mutations on the catalytic activity of DNMT3a3L directly in an intracellular, nuclear environment. We further demonstrate light‐control of a programmable DNMT‐TALE fusion construct, offering selective methylation of user‐defined DNA sequences, and enabling the deposition of defined 5mC levels via tuning the light dosage. Finally, we deliver first insights into the early events of transcriptome reshaping by light activated de novo methylation. Future studies will focus on dissecting the rate and order of early chromatin‐associated events that follow normal and aberrant DNA methylation by individual full‐length and genome‐integrated DNMTs. This will help to understand how the regulation and perturbation of DNMT functions ultimately control the formation of cellular phenotypes during development and malignant transformation.

## Conflict of interest

The authors declare no conflict of interest.

## Supporting information

As a service to our authors and readers, this journal provides supporting information supplied by the authors. Such materials are peer reviewed and may be re‐organized for online delivery, but are not copy‐edited or typeset. Technical support issues arising from supporting information (other than missing files) should be addressed to the authors.

Supplementary
