# Supplementary material for: Light‐Activation of DNA‐Methyltransferases
Source: Angew Chem Int Ed Engl. 2021 May 5;60(24):13507–12. doi: 10.1002/anie.202103945 (PMC8251764; doi:10.1002/anie.202103945)
Supplement: Supplementary file 1 — Supplementary [file ANIE-60-13507-s001.pdf]

Angewandte Chemie
Eine Zeitschrift der Gesellschaft Deutscher Chemiker

Supporting Information

Light-Activation of DNA-Methyltransferases

Jan Wolffgramm, Benjamin Buchmuller, Shubhendu Palei, Álvaro Muñoz-López, Julian Kanne, Petra Janning, Michal R. Schweiger, and Daniel Summerer**

anie_202103945_sm_miscellaneous_information.pdf

TABLE OF CONTENTS

SUPPLEMENTARY FIGURES

- Figure S1. Incorporation of ncAA 1 in HEK293T cells. S4
- Figure S2. Expression of GFP (Y39Amber) and correlation to SatIII- pcDNMT in HEK293T cells. S5
- Figure S3. Expression of SatIII-DNMT and SatIII-pcDNMT in HEK293T cells. S6
- Figure S4. MS/MS spectrum of GFP peptide. S7
- Figure S5. Western Blot of pcDNMT3a3L in HEK293T cells. S8
- Figure S6. Cell morphology of SatIII-pcDNMT transfected HEK293T cells with and without light irradiation. S9
- Figure S7. Analysis of flow cytometry data of pcDNMT3a3L activity in HCT116 DKO cells.S10
- Figure S8. Comparison of activity of pcDNMT3a3L in HCT DKO cells with different expression levels. S11
- Figure S9. Global methylation by wt and mutant DNMT3a3L in HCT116 DKO cells. S12
- Figure S10. Expression level of wt and mutant pcDNMT3a3L in HCT116 DKO cells. S13
- Figure S11. Titration of ncAA 1 in HEK293T cells. S14
- Figure S12. Heat shock response of SatIII-pcDNMT expressing HEK293T cells. S15
- Figure S13. SatIII methylation levels in SatIII-DNMT, SatIII-pcDNMT expressing or untransfected HEK293T cells. S16
- Figure S14. Methylation of seconds CpG of SatIII locus by SatIII-pcDNMT in HEK293T cells. S17
- Figure S15. On- and off-target Sanger sequencing. S18
- Figure S16. Methylation of p16 locus with p16-DNMT in HEK293T cells. S19
- Figure S17. Decaging kinetics of 1 (1 mM in PBS). S20
- Figure S18. Clustering of differentially expressed genes in pcDNMT3a transfected HEK293T cells. S21
- Figure S19. Plasmid map of pJaW911. .... S43
- Figure S20. Plasmid map of pJaW1660. .... S43
- Figure S21. Plasmid map of pJaW1998. .... S44
- Figure S22. Plasmid map of pStH1169. .... S44
- Figure S23. Plasmid map of pJaW1849. .... S45

MATERIAL AND METHODS

- Vector Construction S22
- Light-controlled global DNA methylation in HCT116 (DKO) cells S23
- Analysis of FACS data with R S24
- Targeted, light controlled DNA methylation and microscopy in HEK293T cells S24
- Imaging of HSF1 and TALE-DNMT in HEK293T cells S24
- Sorting, gDNA isolation and bisulfite conversion S25
- Pyrosequencing analysis S25

- Sanger Sequencing S25
- Illumina Sequencing S25
- RNA extraction and transcriptome analysis by RNA-seq S26
- Western Blot S27
- Mass Spectrometry S27
- Synthesis of 1 (4,5-Dimethoxy-2-Nitrobenzyl-L-Cysteine) S28

SUPPLEMENTARY TABLES

- Table S1. Quickchange primer for DNMT mutations. S23
- Table S2. Primary Antibodies S29
- Table S3. Secondary Antibodies S29
- Table S4. Oligonucleotides for cloning. S29
- Table S5. Oligonucleotides for bisulfite PCR. S30
- Table S6. Oligonucleotides for RT-qPCR. S31
- Table S7. Mutation table of COSMIC database with frequencies S32
- Table S8. Sample assignments for SatIII bisulfite-converted amplicons. S32
- Table S9. Read counts of SatIII bisulfite-converted amplicons by analysis stage. S32
- Table S10. Methylation levels of both SatIII CpGs. S33
- Table S11. Description of RNA sequencing samples and mapping statistics. S34
- Table S12. Number of genes reported differentially expressed upon light-induction after 4h and 8h. S34
- Table S13. Log2-fold changes of genes after 4h. S34
- Table S14. Log2-fold changes of genes after 8h. S36
- Table S15. Overall differential gene expression after light-induction. S37

SUPPLEMENTARY REFERENCES S40

APPENDIX

- Sequence DNMT3a3L S42
- SatIII Sequence S42
- Plasmid Maps S43

SUPPLEMENTARY FIGURES

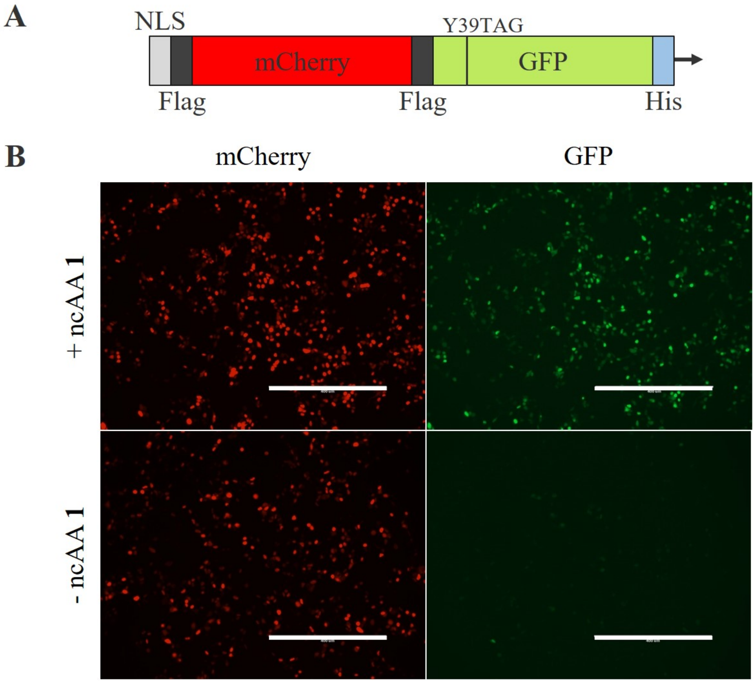

Figure S1. Incorporation of ncAA 1 in HEK293T cells.

A) Design of the suppression control with nuclear localization signal (NLS), Flag-Tags and His-Tag. **B)** Selective incorporation of ncAA **1** in HEK293T resulting in GFP expression 24h after transfection. Scale bar is 400 µm.

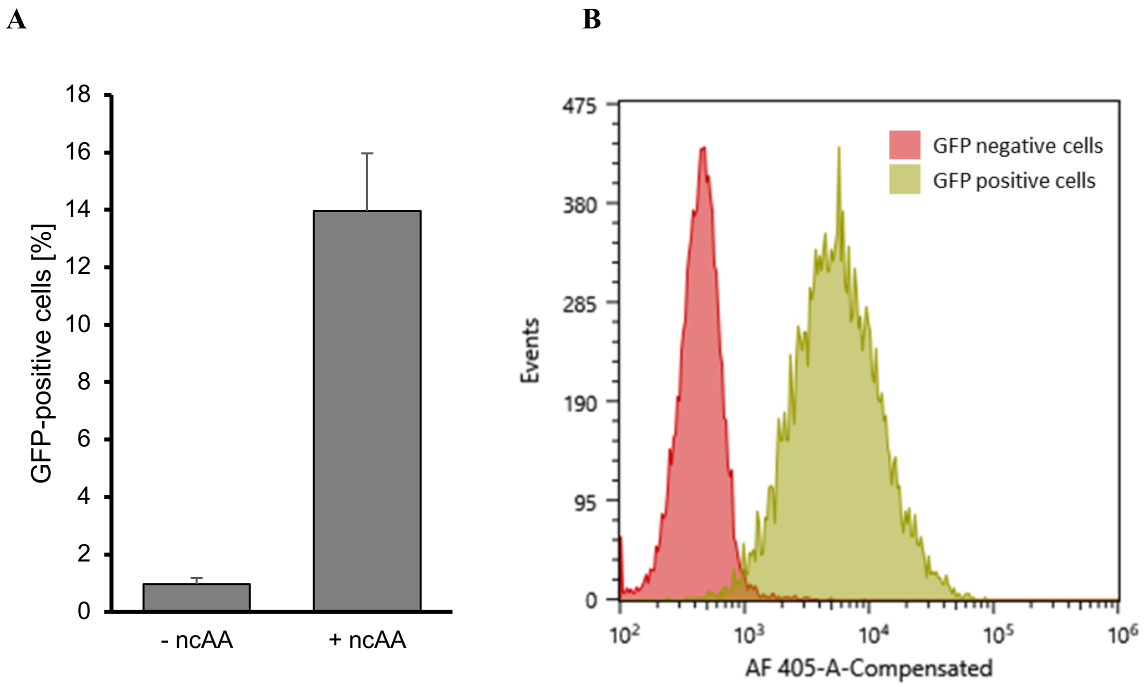

Figure S2. Expression of GFP (Y39Amber) and correlation to SatIII- pcDNMT in HEK293T cells. A) Selective expression of GFP only in the presence of ncAA **1** 24h after transfection. Error bars show standard deviations from two independent biological replicates. **B)** Histograms of SatIII-pcDNMT expression levels (staining of HA-tag with AF405) in GFP negative and positive cells. The expression of SatIII-pcDNMT is correlated with the expression of GFP, allowing the use of the GFP transfection control for sorting of live cells expressing SatIII-pcDNMT for downstream methylation analyses.

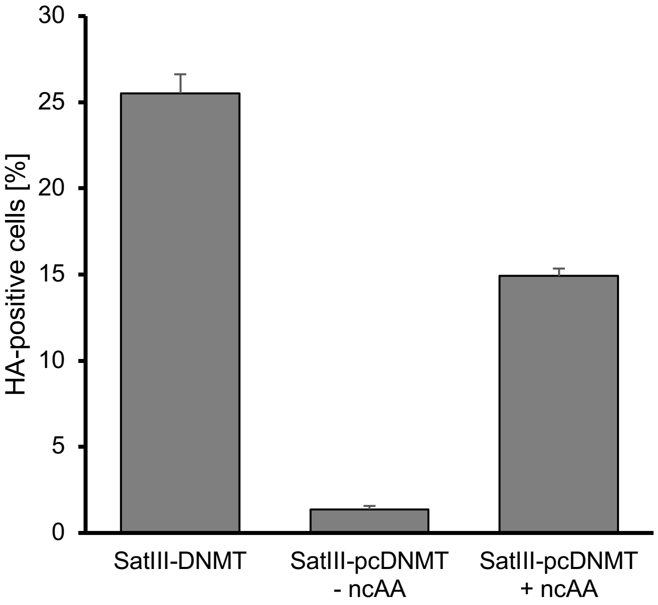

Figure S3. Expression of SatIII-DNMT and SatIII-pcDNMT in HEK293T cells.

Selective amber suppression in SatIII-pcDNMT only in the presence of 0.05 mM ncAA **1** 24h after transfection. Cells were stained with anti-HA primary and fluorescein labeled secondary antibody. Error bars show standard deviations from three independent biological replicates.

A

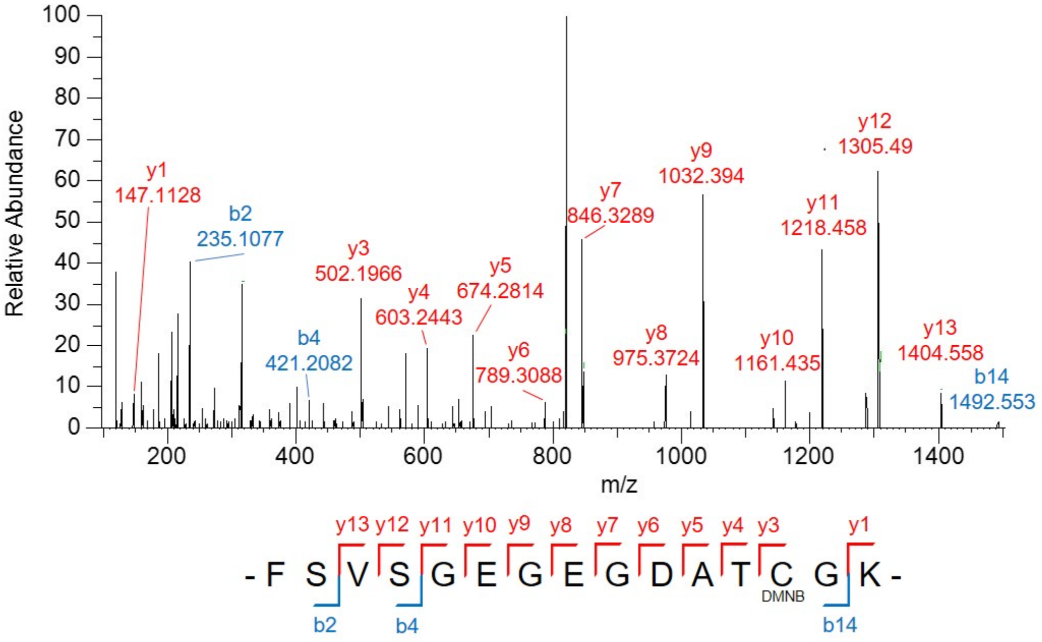

B

Sample	Cysteine modification	Theoretical mass	Measured mass
Caged without light	DMNB	1639.6660	1639.6637

Figure S4. MS/MS spectrum of GFP peptide.

Detected fragments of the peptide -FSVSGEGEGDATCGK- from the transfection control mCherry-GFP(Y39Amber) covering the amber site used for incorporation of DMNB-Cys in HEK293T cells. **A)** Spectrum of the photocaged (DMNB) peptide w/o light. **B)** Comparison of theoretical mass and measured mass.

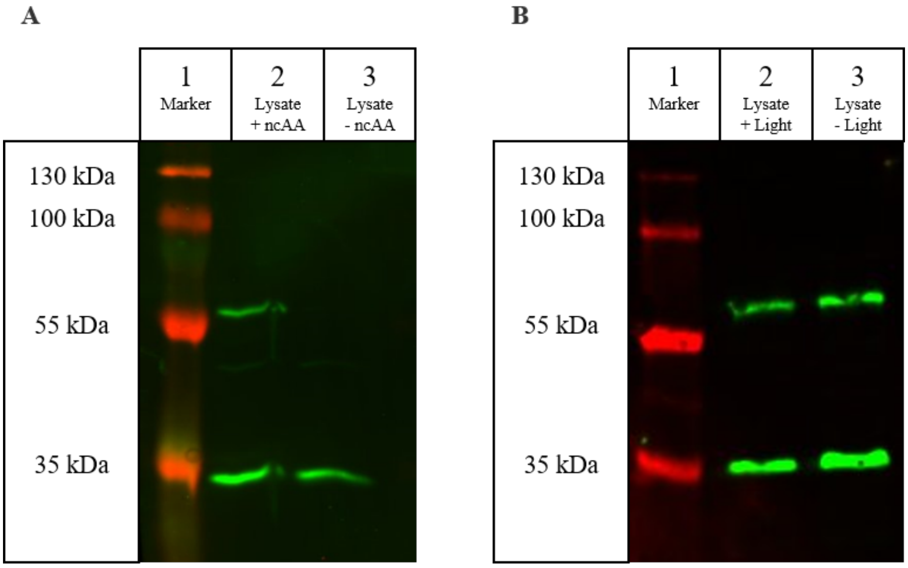

Figure S5. Western Blot of pcDNMT3a3L in HEK293T cells.

Lysates from pcDNMT3a3L-transfected HEK293T cells grown in the presence or absence of 0.5 mM ncAA **1** **(A)** or lysates from pcDNMT3a3L-transfected HEK293T cells grown in the presence of 0.05 mM ncAA **1** and irradiated or not irradiated with light **(B)** were used for Western Blot analysis. GAPDH-antibodies were used as internal control, and HA-antibodies for pcDNMT3a3L detection. The expected sizes (35.9 kDa for GAPDH and 64.9 kDa for pcDNMT3a3L) were detected, indicating full length expression of pcDNMT3a3L only in the presence of ncAA **1**. As reference, the PageRuler™ Plus Prestained Protein Ladder was used.

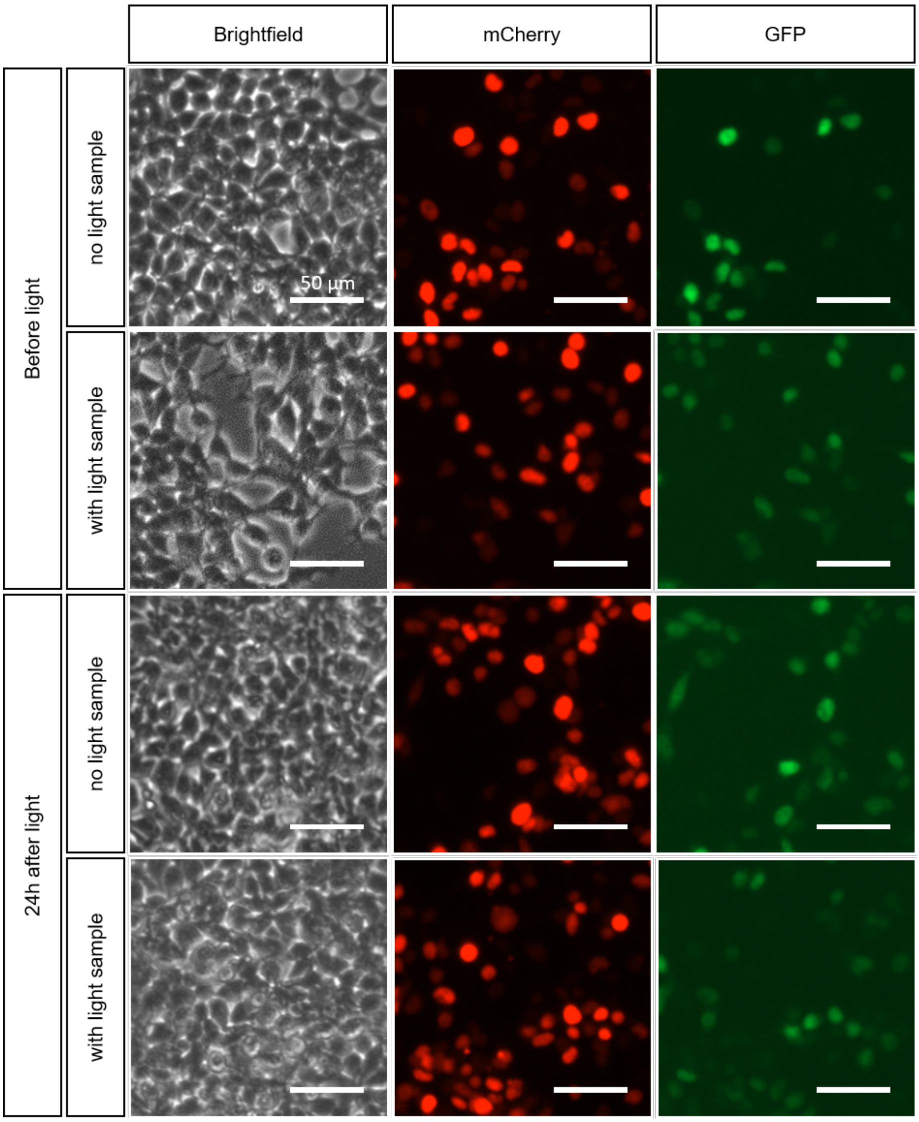

Figure S6. Cell morphology of SatIII-pcDNMT transfected HEK293T cells with and without light irradiation.

Pictures were taken directly before and 24 h after 5 min light irradiation for the 'light' and 'no light' samples. The plasmid also carried the transfection control gene. Scale bar is 50 µm.

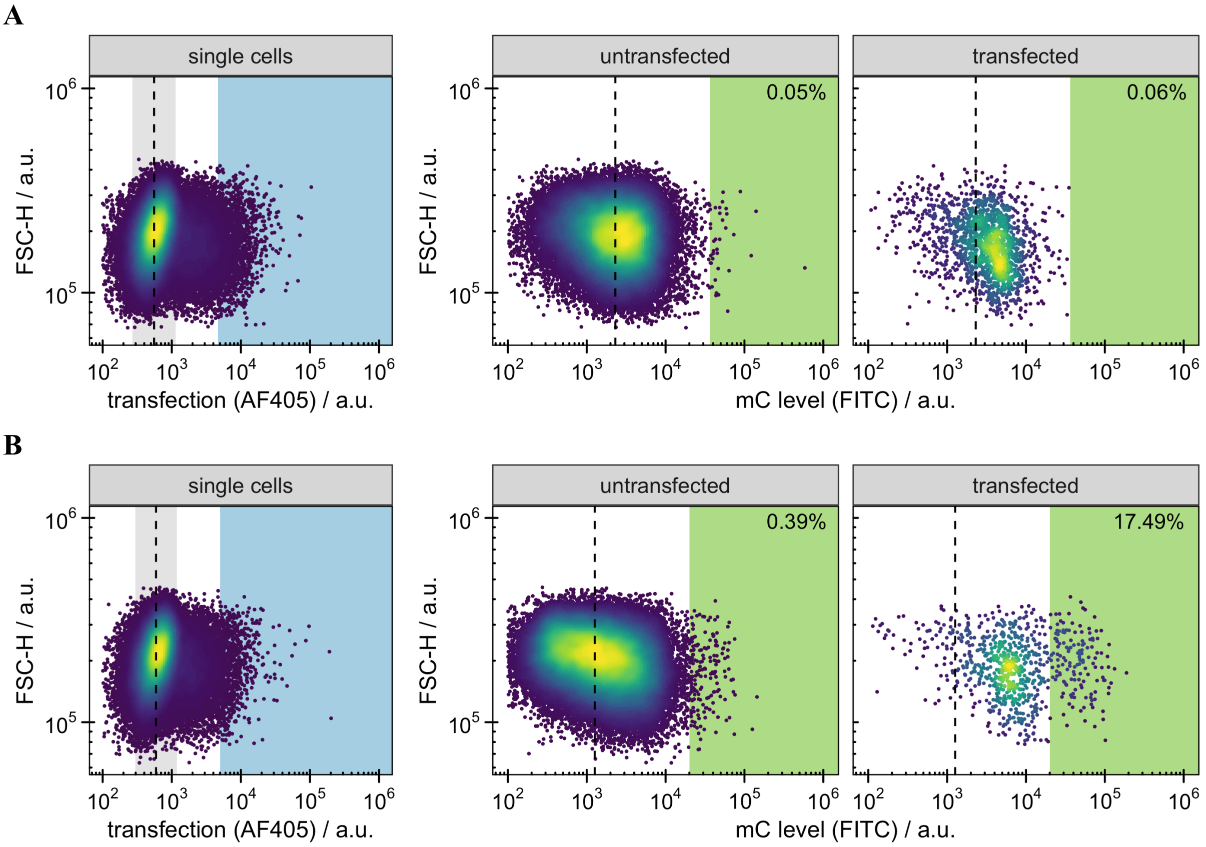

Figure S7. Analysis of flow cytometry data of pcDNMT3a3L activity in HCT116 DKO cells. Representative images of flow cytometry data analyzed with R. Shown is one replicate of wild type pcDNMT3a3L without **(A)** and with **(B)** light activation. In the first plot, the blue gate describes the transfected cells (based on HA stain with AF405) and its threshold is defined as 8.4 times above the median of untransfected cells. The green gate in the next plot describes the 5mC positive cells (based on 5mC stain with FITC) and its threshold is defined as 15.4 times above the median of untransfected cells.

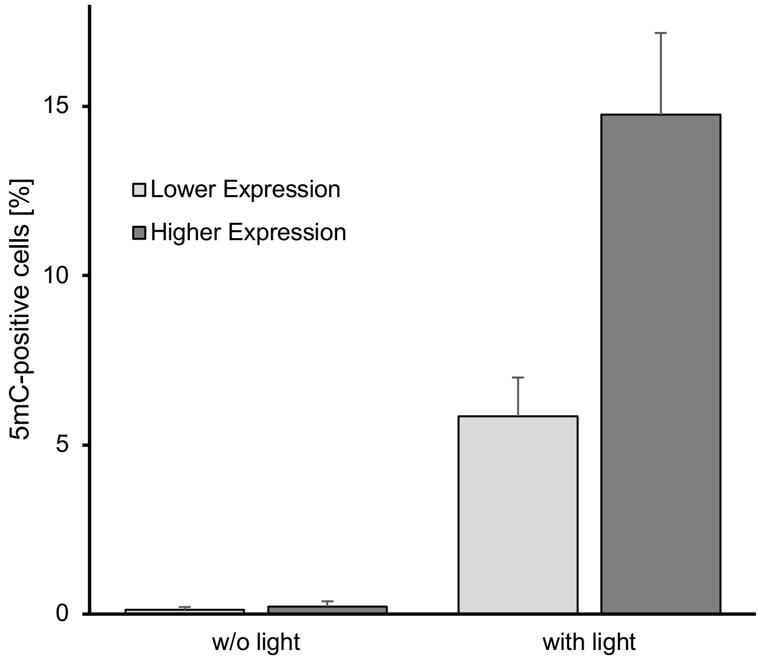

Figure S8 Comparison of activity of pcDNMT3a3L in HCT DKO cells with different expression levels.

HCT DKO cells transfected with pcDNMT3a3L were either grouped into "lower expression" or "higher expression" based on their HA immunostaining of pcDNMT3a3L. Lower expression was defined as 6 to 8 times the median and higher expression with a threshold of 8.4 times above the median of HA immunostaining of untransfected cells. Error bars show standard deviations from three independent biological replicates.

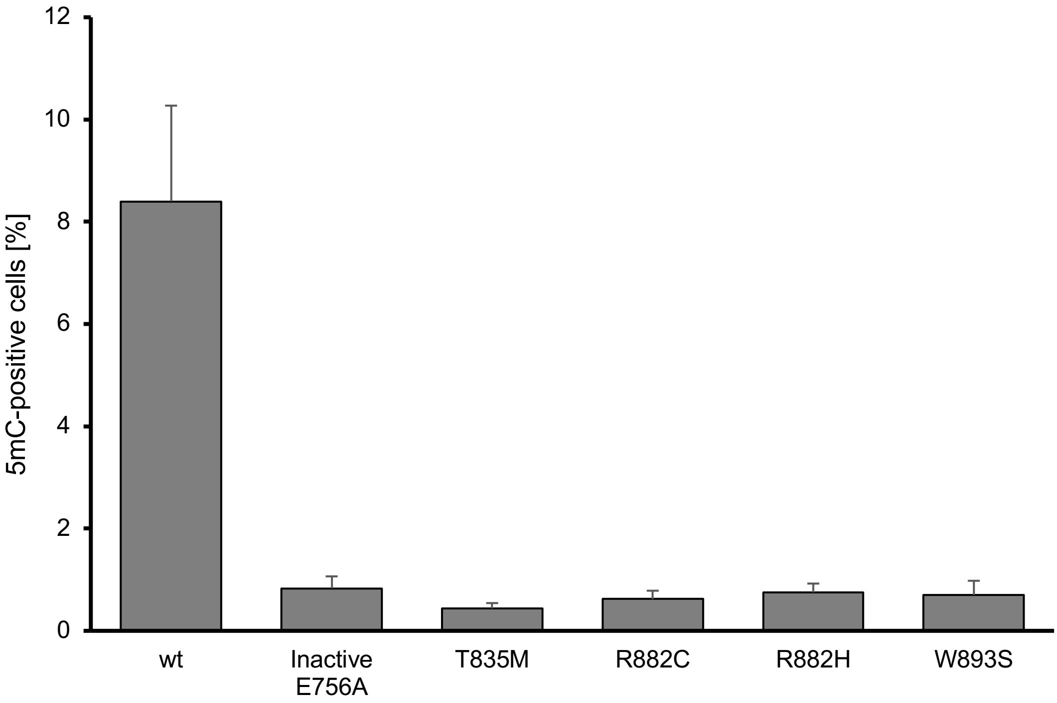

Figure S9. Global methylation by wt and mutant DNMT3a3L in HCT116 DKO cells.

FCM analysis of cells expressing wild type or mutant DNMT3a3L. Cells were immunostained with HA- and 5mC-antibodies. Error bars show standard deviations from three independent biological replicates.

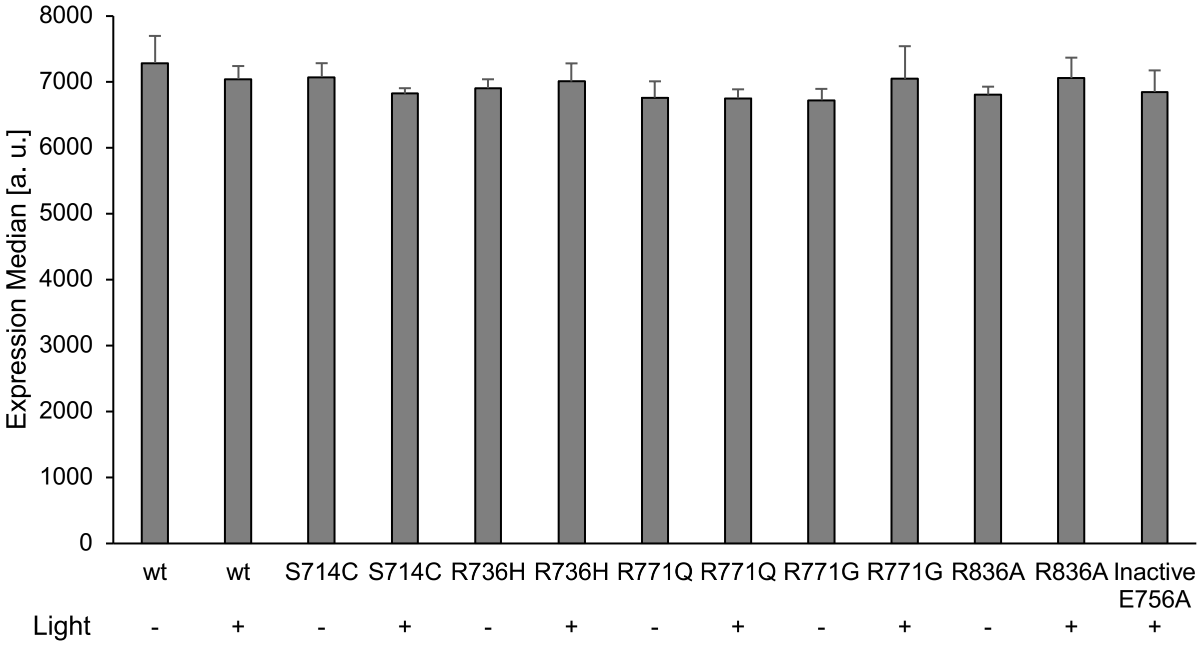

Figure S10. Expression level of wt and mutant pcDNMT3a3L in HCT116 DKO cells.

Median expression level of pcDNMT3a3L constructs corresponding to Fig. 2c based on immunostaining against the C-terminal HA-tag. Error bars show standard deviations from three independent biological replicates.

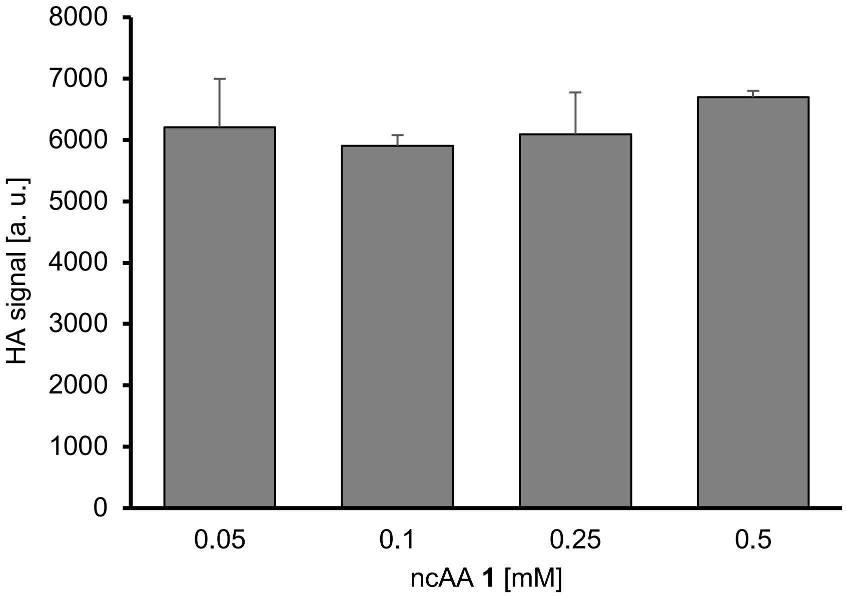

Figure S11. Titration of ncAA 1 in HEK293T cells.

Average HA signal intensity of SatIII-pcDNMT expressing HEK293T cells (anti-HA immunostain with AF405). Error bars show standard deviations from two independent biological replicates.

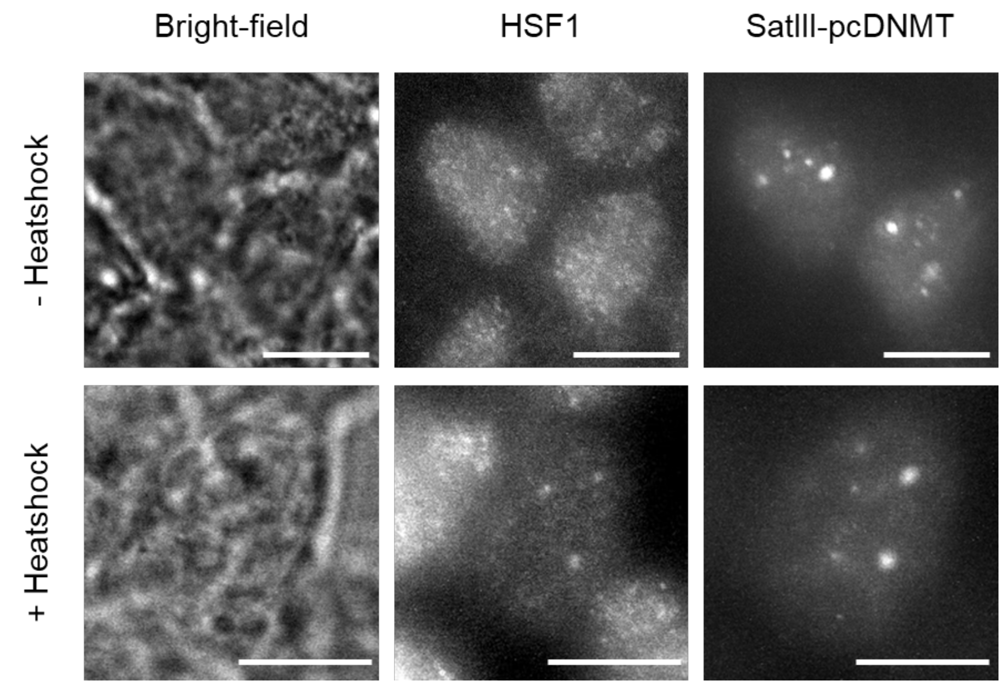

Figure S12. Heat shock response of SatIII-pcDNMT expressing HEK293T cells.

Co-localization of HSF1 and SatIII-pcDNMT in heat-stressed (1 h 44 °C) HEK293T cells immunostained with anti-HA and anti-HSF1 antibodies. Scale bar is 10 μm.

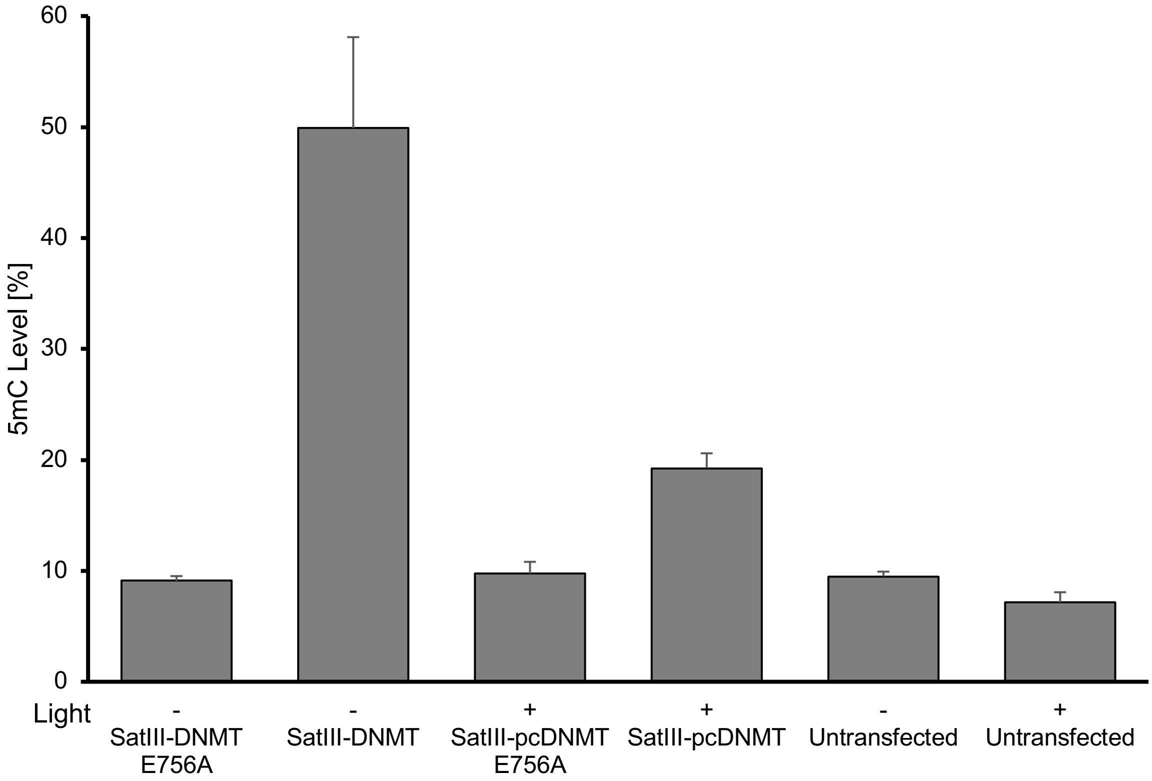

Figure S13. SatIII methylation levels in SatIII-DNMT, SatIII-pcDNMT expressing or untransfected HEK293T cells.

Methylation of SatIII locus by SatIII-DNMT 24h after transfection and SatIII-pcDNMT 24h after 5 min light irradiation and the controls of their inactive E756A variants measured by bisulfite PCR and pyrosequencing.[1] For SatIII-pcDNMT, cells were irradiated 24 h after transfection. A heat shock (1 h 44 °C) was done for all samples prior to analysis. Error bars show standard deviations from two independent biological replicates.

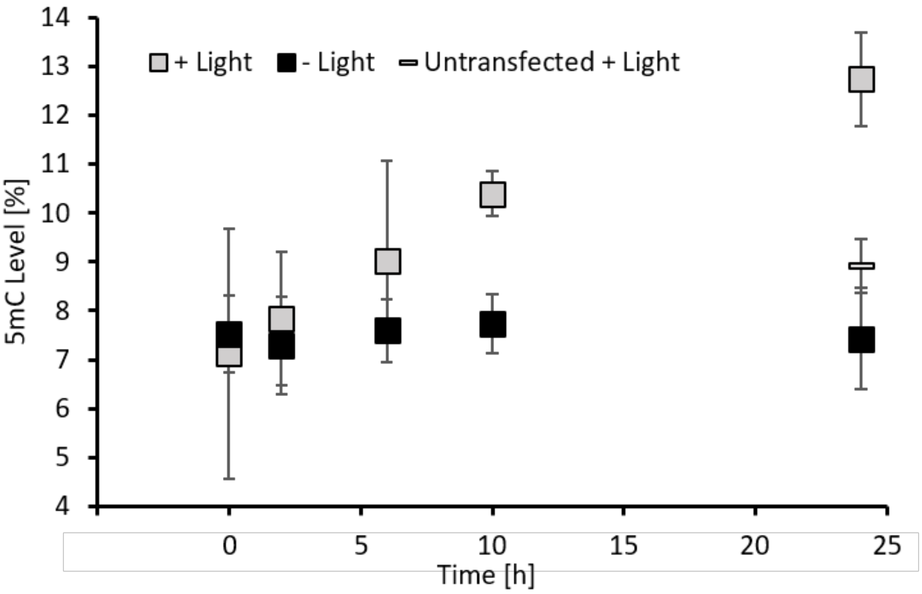

Figure S14. Methylation of second CpG of SatIII locus by SatIII-pcDNMT in HEK293T cells. 5mC level of the second CpG of the SatIII locus near the binding site of SatIII-pcDNMT in HEK293T cells analyzed by Illumina sequencing. Cells were irradiated with 5 min light 24 h after transfection with SatIII-pcDNMT and sorted at indicated times later. gDNA was bisulfite converted and amplified SatIII locus was analysed by Illumina sequencing. As controls, untransfected cells were also irradiated with light and analysed 24 h later. Additional, controls without light were done for all time points. Error bars show standard deviations from three independent biological replicates. For an explanation of CpG positions, see Appendix (paragraph "SAT III sequence").

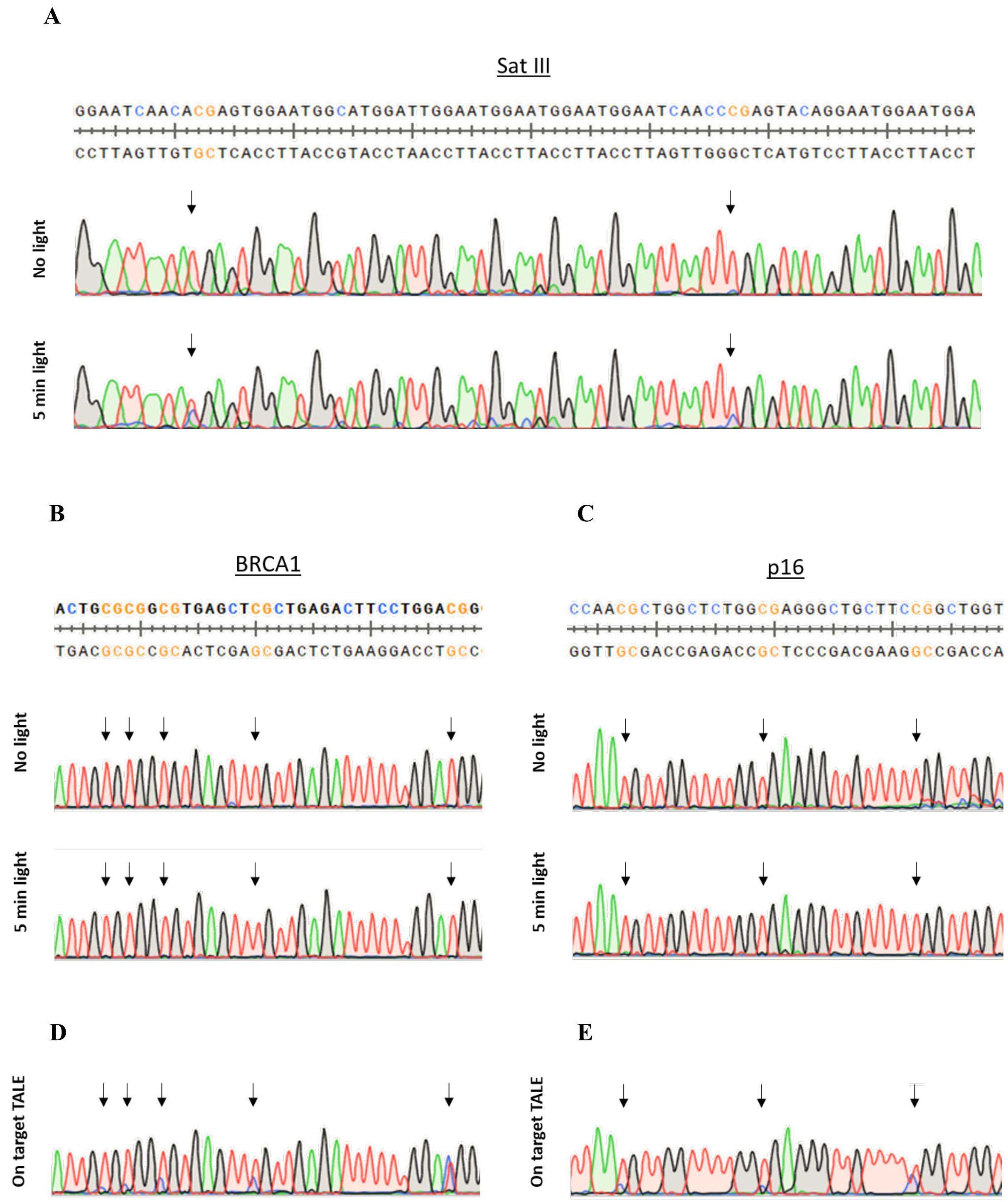

Figure S15. On- and off-target Sanger sequencing.
Methylation of SATIII (**A**), BRCA1 (**B**) and p16 (**C**) by SATIII-pcDNMT 24 h after 5 min light or on target non-photocaged constructs for BRCA1 (**D**) and p16 (**E**).

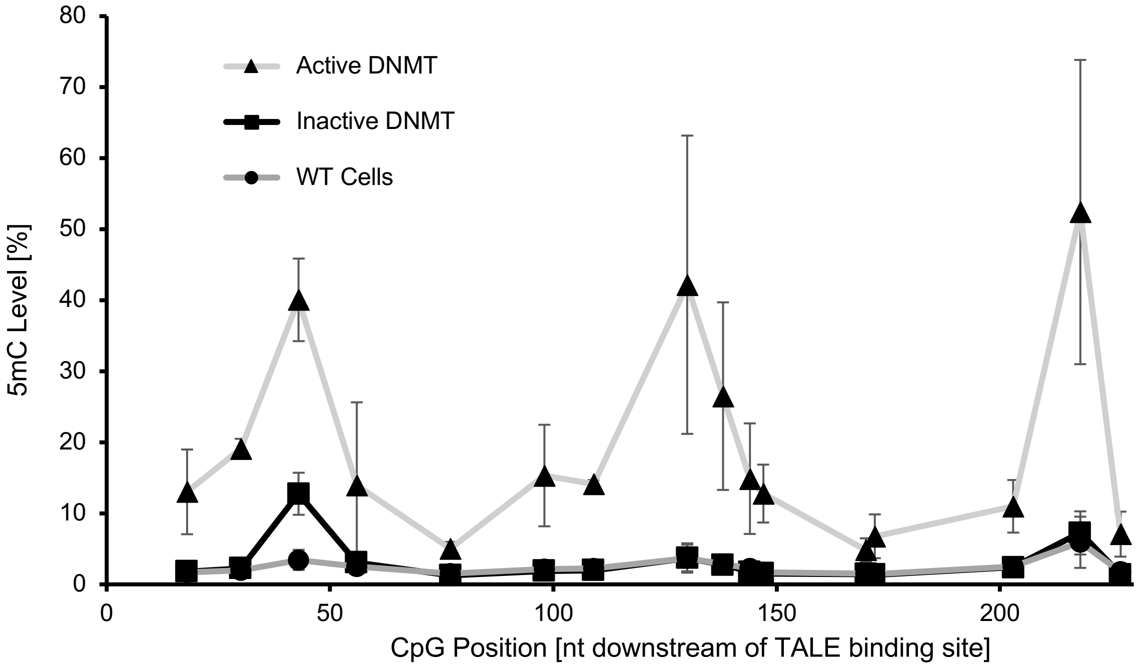

Figure S16. Methylation of p16 locus with p16-DNMT in HEK293T cells.

5mC level of the CpGs 18 to 227 nt downstream of the p16-DNMT binding site in HEK293T cells analyzed by Illumina sequencing in independent duplicate experiments for DNMT, inactive DNMT E756A and untransfected (WT) cells. Note that the target sequence of p16-DNMT is identical to one previously targeted with a TALE-DNMT construct and shows similar methylation profile.[2] Numbering starting with 5-T of TALE target. Error bars show standard deviations from two independent biological replicates.

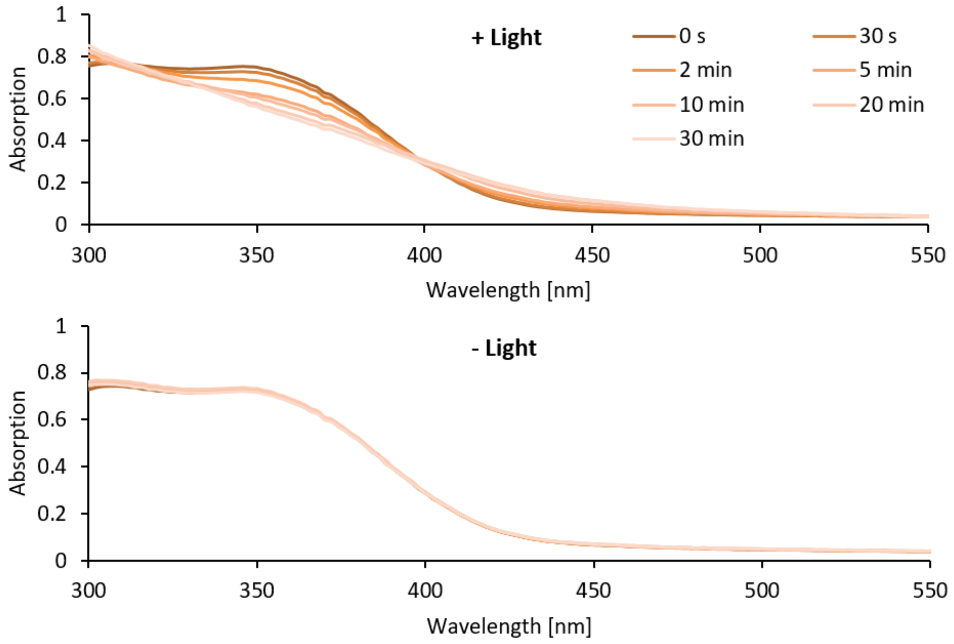

Figure S17. Decaging kinetics of 1 (1 mM in PBS).

Decaging with subsequent absorption measurement was conducted with indicated irradiation times under identical conditions as all cellular decaging experiments within this study.

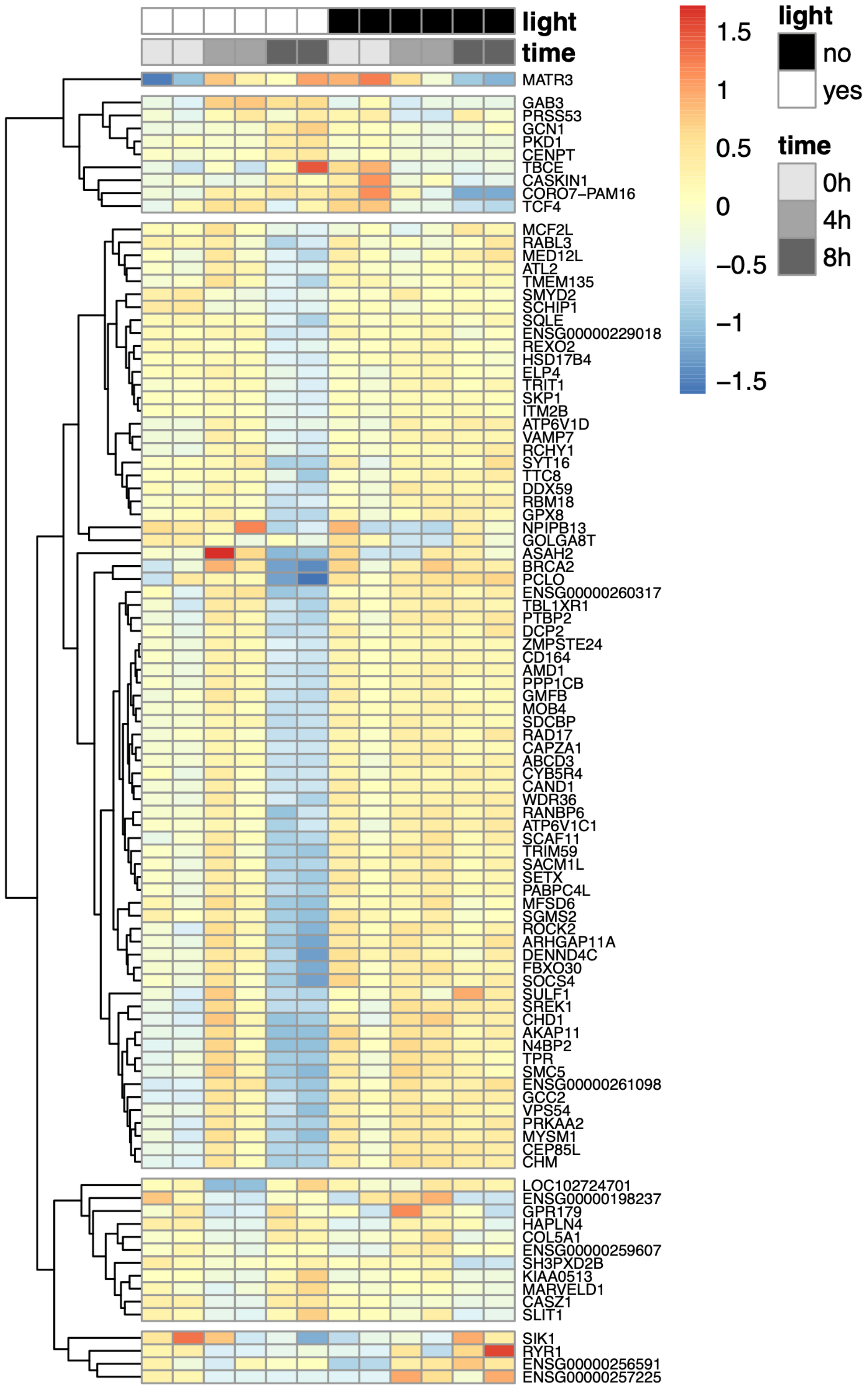

Figure S18. Clustering of differentially expressed genes in pcDNMT3a transfected HEK293T cells.

Total RNA was isolated and sequenced 0 h, 4 h and 8 h after light irradiation. Additional the respective controls without light were analyzed. Shown genes have a *p*-value of 0.05 and their difference of regularized log2-normalized counts of the mean expression of all samples is shown as a color code.

MATERIAL AND METHODS

Vector Construction

All enzymes were purchased from New England Biolabs. Plasmid maps can be found in the appendix.

Golden gate 2 entry plasmid pJaW911 was cloned by Gibson assembly [3]. For this, plasmid pAnW891 (based on Addgene plasmid #47389) was cut with PacI and AscI to remove the VP64 gene and mixed with the insert DNMT3a3L which was amplified from plasmid pET28-DNMT3a3L-sc27 (Addgene #71827) using primers o2071/o2072. Mutations for the catalytically inactive DNMT3a3L E756A or DNMT3a3L C710Amber (further “pcDNMT3a3L”) were introduced by Quickchange site directed mutagenesis using primer pairs o2038/o2039 or o2175/o2176 respectively, resulting in plasmids pJaW915 (DNMT3a3L E756A), pJaW1038 (pcDNMT3a3L) and pJaW1041 (pcDNMT3a3L E756A).

TALEs were assembled according to a previously published protocol[4] using all previously described entry plasmids in golden gate 2 reactions, resulting in plasmids coding for the SATIII-targeting TALE protein (RVD sequence: NN NI NG NG HD HD NI NG NG HD HD NI NG NG HD HD NI NG NG) followed by wt (pJaW1648), inactive E756A (pJaW1654) or C710Amber (active: pJaW1660; inactive: pJaW1666) DNMT3a3L and a C-terminal HA tag. Additional, TALEs to target the p16 gene (HD HD NG HD HD NG NG HD HD NG NG NN HD HD NI NI HD NN HD NG NN NN HD NG)[2] and the BRCA1 gene (HD NI NN NN NI NN NN HD HD NG NG HD NI HD HD HD NG HD NG NN HD NG HD NG NN) were assembled into pJaW911 resulting in pJaW926 and pJaW1002, respectively, and for p16 also in pJaW915 (inactive DNMT3a3L E756A) resulting in pJaW935.

The orthogonal *E. coli* leucyl-tRNA-synthetase (ecLRS-BH5) with five previously reported mutations M40G, L41Q, Y499L, Y527G, H537F[5] and the suppressor $tRNA_{CUA}$ ($Leu5_{CUA}$)[6] were encoded on plasmid pStH1147. For cloning, the synthetase was amplified with primer pair o2332/o2333 from plasmid pescLRSNVOCBH5-4 (pDas49) and the backbone from plasmid pCMV_PylRS_AF (pDaS273) with primer pairs o2330/o2331 and o2334/2335. Further, the $tRNA_{CUA}$ was obtained by hybridizing oligonucleotides o2336/o2337, and all four fragments were mixed for Gibson assembly. To increase the fidelity of ecLRS-BH5, the mutation T252A[5] was introduced by Quickchange site directed mutagenesis using primer pair o2475/o2476, affording pStH1169.

To obtain plasmid pJaW1131 (encoding global DNMT3a3L) the golden gate 2 entry site was removed by Gibson assembly after amplifying the backbone of pJaW911 with primers o2253/o2254. Further, mutations for inactive and amber variants were introduced as before, resulting in inactive DNMT3a3L E756A (pJaW1580), pcDNMT3a3L (pJaW1559) and inactive pcDNMT3a3L E756A (pJaW1775) with C-terminal HA tag.

To avoid possible light bleed-through in microscopy and to decrease the need for compensation in flow cytometry the transfection control (mCherry-Y39Amber-GFP) was removed from plasmids pJaW1038 and pJaW1041 by Gibson assembly after amplifying two parts of the backbone with primer pairs o3352/o2434 and o2433/o3355. Afterwards, the SatIII-TALE was assembled into both plasmids as described before resulting in plasmids pJaW1998 (SatIII-pcDNMT) and pJaW1999 (inactive SatIII-pcDNMT E756A).

The transfection control was also removed from all global DNMT plasmids by Gibson assembly and amplification of the backbone with primer pairs o3352/o2435 and o2436/o3355 resulting in plasmids pJaW1848 (DNMT3a3L), pJaW1850 (inactive DNMT3a3L E756A), pJaW1849 (pcDNMT3a3L) and pJaW1851 (inactive pcDNMT3a3L E756A).

DNMT mutations were introduced on plasmids pJaW1848 or pJaW1849 by Quickchange site directed mutagenesis with following primer pairs:

Table S1. Quickchange primer for DNMT mutations.

Mutation	Primer pair	Resulting plasmid (w/o C710Amber)	Resulting plasmid (with C710Amber)
S714C	o3672/o3673	---	pJaW1978
R736H	o3674/o3675	---	pJaW1979
R771Q	o3676/o3677	---	pJaW1980
R771G	o3678/o3679	---	pJaW1981
T835M	o3680/o3681	pAlH1902	---
R836A	o3684/o3685	---	pJaW1984
R882C	o3686/o3687	pAlH1905	---
R882H	o3688/o3689	pAlH1906	---
W893S	o3690/o3691	pAlH1907	---

For further studies, the 3L part from plasmid pJaW1131 was deleted resulting in plasmid pJaW2557. The deletion was done by Gibson cloning by amplifying pJaW1131 with primers o3569/o3574 and o3575/o3570. Further mutagenesis for C710Amber variant was done as described before, resulting in pJaW2559 (pcDNMT3a).

Light-controlled global DNA methylation in HCT116 (DKO) cells

400.000 HCT116 DKO cells (DNMT1 (Δexons3-5/Δexons3-5), DNMT3B (-/-), Horizon Discovery Ltd., product number HD R02-022; negatively tested for mycoplasma infection) were seeded on a 3.5 cm dish (Sarstedt #83.3900) in 2 mL normal growth medium (RPMI 1640 (with L-Glu) + 10% FBS + 1% Pen/Strep) and incubated at 37 °C and 5 % CO_2. On the next day, 3 µL X-tremeGENE9 (Sigma) per 1000 ng plasmid DNA was mixed with 150 µL OptiMEM (Gibco). Also, plasmid DNA (2 µg pcDNMT3a3L plasmid and 1 µg p1169) was mixed in additional 150 µL OptiMEM and both mixtures were combined after 5 min incubation at RT and incubated for additional 20 min. Then, 4,5-dimethoxy-2-nitrobenzyl-L-cysteine (**1**) was added to the transfection mix at a final concentration of 0.05 mM, and the transfection mixture was added drop-wise to the cells. After 48h the medium was replaced with 1 mL 37 °C preheated DPBS. Cells were placed on a 365 nm UV-transilluminator (Witeg DH.WUV00010, 6x 15 W) for 5 min. After light-exposure, the DPBS was replaced immediately with 2 mL fresh and preheated growth medium w/o **1**, and cells were incubated for further 24 h. Cells were washed with DPBS and trypsinized (0.05% / EDTA 0.02% (PAN Biotech)) for 4 min at 37 °C. After blocking the reaction with 1 mL growth medium, cells were washed in 3 mL DPBS (centrifuged 5 min 500 rcf) and then fixed with 100 µL medium A (Fix & Perm Kit, Thermo Fisher Scientific) for 15 min at RT. After washing with 2 mL wash buffer (PBS + 5% FBS + 0.1% NaN_3), cells were permeabilized with 100 µL medium B for 20 min and then washed again. To denature chromosomal DNA, cells were incubated for 20 min with 2 N HCl, centrifuged and resuspended in 1 mL DPBS (15 min incubation). Cells were blocked with 1 mL blocking buffer (PBS + 1% BSA + 0.05% Tween) shaking overnight at 4 °C. C-terminal HA-Tag and 5mC were immunostained with rabbit anti-HA and mouse anti-5-methylcytosine primary antibodies (both diluted 1:400 in 100 µL blocking buffer) shaking for 1 h at RT. After washing twice with PBS-T (0.05 % Tween-20), cells were incubated with secondary antibodies (diluted 1:400 in 100 µL blocking buffer) for 1 h at RT. Then, cells were washed twice with PBS-T, once with DPBS and measured by flow cytometry (Sony SH800S Cell Sorter with 405 nm, 488 nm, 561 nm and 638 nm lasers with manufactures optical filter pattern 2).

Analysis of FACS data with R

Flow cytometry data of single cells were exported with Cell Sorter Software (Sony Biotechnology) to a comma-separated values file and analyzed with R (v. 3.6.2)[7] using the data.table package[8] and visualized using ggplot2[9]. Untransfected cells were selected as the most abundant unstained population from a Gaussian mixture model[10]. Transfected cells were defined with respect to a threshold of 8.4 times above the median of untransfected cells in the HA stain. Of such cells, 5-methylcytosine positive cells were defined with a threshold of 15.85 times the median of the untransfected cells under the same conditions in the 5mC stain (see figure S4).

Targeted, light controlled DNA methylation and microscopy in HEK293T cells

600.000 HEK293T cells (negatively tested for mycoplasma infection) were seeded on a 3.5 cm dish (Sarstedt #83.3900) or on µ-Dish 35 mm plates for microscopy (Ibidi; coated with 0.01 % poly L-lysine in DPBS for 1 h at 37°C) in 2 mL normal growth medium (DMEM (w/o L-Glu) + 10% FBS + 1% Pen/Strep + 1% L-Glu). On the next day, 3 µL FuGENE6 (Promega) or PEI per 1000 ng plasmid DNA were mixed with 100 µL OptiMEM and incubated for 5 min at RT. Then, 2 µg TALE-pcDNMT plasmid and 1 µg p1169 was mixed by pipetting and incubated for another 15 min. 0.05 mM ncAA **1** was additionally added to the transfection mix and then added drop-wise to the cells. 24 h after transfection, light irradiation was performed as described before and cells were incubated for further 24 h. For heat shock experiments, cells were incubated at 44 °C for 1 h prior to analysis.

Imaging of HSF1 and TALE-DNMT in HEK293T cells

HEK293T were exposed to heat stress by incubation at 44 °C for 1 h. Then, cells were fixed by adding formaldehyde to a final concentration of 1 % to the medium. After 5 min incubation at 37 °C, cells were washed with DPBS and then incubated for 15 min at RT in 1 mL DPBS + 0,25 % Triton X-100. After washing once, cells were blocked in 1 mL DPBS + 1% BSA + 0.05 % Tween-20 for 30 min at RT. Staining was done by adding 500 µL DPBS with 0.625 µL HA (mouse) and HSF1 (rabbit) antibody each. After 2 h incubation at RT, cells were washed with PBS-T and the secondary antibodies goat anti mouse AF488 und goat anti rabbit Cy5 (each 1:800 diluted) were added. After 1 h at RT, cells were washed again with PBS-T and then stored in 2 mL DPBS for microscopy. Images were taken as z-stacks of FretGFP and Cy5 channels using a 60X oil objective with an Olympus IX81 microscope coupled with Hamamatsu model C10600-10B-H camera with an exposure time of 10 ms each. The subcellular localization of foci was analyzed from maximal intensity Z-projections of image stacks using the Fiji distribution of ImageJ.[11]

Sorting, gDNA isolation and bisulfite conversion

HEK293T cells were washed with 1 mL DPBS and trypsinized (500 µL, 4 min 37 °C). The cell suspension was transferred into 4 mL DPBS and centrifuged (5 min, 500 rcf). The pellet was resuspended in 300 µL 4 °C cold DPBS and kept on ice afterwards. Sorting for high mCherry or high GFP (for TALE-pcDNMT constructs) expressed cells was done with a SONY SH800S cell sorter. Sorted cells were centrifuged as before, and the supernatant was removed to a residual volume of 200 µL. Genomic DNA was isolating using the QIAamp DNA Mini Kit (Qiagen #51304) according to the manufacturers protocol. Subsequent bisulfite conversion was conducted with the EpiTect Bisulfite Kit (Qiagen #59104) according to the manufactures protocol.

Pyrosequencing analysis

Pyrosequencing analysis was done as previously reported.[12]
Briefly, the SatIII locus was amplified from bisulfite-converted gDNA using the EpiTect MSP Kit (Qiagen #59305) and primers o3266/o3439. Then, the PCR product was prepared for sequencing and mixed with sequencing primers as reported. The pyrosequencing reaction was performed in a PSQ HS 96ATwo Pyrosequencer and analysed using the PSQ HS 96A software.

Sanger Sequencing

To analyze on and off target effects, SatIII, BRCA1 and p16 loci were amplified from bisulfite-converted gDNA using the Qiagen MSP Kit and primer pairs o3268/o3269, o2348/o2220 and o2344/o2800 respectively. In a second PCR the product was used as template and amplified using Taq polymerase, o2363 as forward primer and the previously used reverse primers. After column purification (NucleoSpin Gel and PCR Clean-up Kit, Macherey-Nagel #740609.250), amplicons were sequenced using o2363 (SatIII), o2220 (BRCA1) or o2199 (p16). For the BRCA1 locus targeted with BRCA1-DNMT, a slightly different protocol was used. The first PCR with primers o2348/o2220 was used as template in a second PCR (Qiagen MSP Kit) with primers o2350/o2220. This product was then used as template for a third PCR using Taq polymerase and primers o2370/o2220. Purification and sequencing was performed as described before.

Illumina Sequencing

To measure the on target methylation by p16-DNMT, HEK293T cells were transfected with 2 µg pJaW926 or pJaW935 and sorted for mCherry 48h later. gDNA was isolated and bisulfite converted as described before. Amplification of the p16 locus downstream of the TALE binding site was done using the Qiagen MSP Kit and primer pairs o2339/2199 and o2340/2201. A barcode was added by using the same reverse primer in a second PCR using Taq polymerase and o2363 or o2366 as forward primer for pJaW926 or pJaW935 samples, respectively. Library preparation and Illumina sequencing was done by GATC (2x 150 bp paired end) and analysis was conducted using Qiagen Genomic Workbench.
For the monitoring of SatIII 5mC-levels, HEK293T cells were transfected with SatIII-pcDNMT in the presence of 0.05 mM DMNB-Cys as described before. 24 h later, the time course started with 5 min light irradiation and transfected cells were sorted at the respective time points. After gDNA isolation and bisulfite conversion, SatIII

locus was amplified with the Qiagen MSP Kit and primer pair o3268/o3269. DNA barcodes were added in a second PCR using Taq polymerase and different primer pairs according to Table S8. Barcoded amplicons were pooled in two sequencing runs (Table S8) and sent for next-generation sequencing on an Illumina Miseq platform (Genewiz, Leipzig, Germany, Amplicon-EZ service). The trimmed 250-mer pairs were merged with Pandaseq v. 2.11[13] using a k-mer length of 3. The merged reads were then mapped against the *in silico* bisulfite-converted sequence of the original top strand (see appendix SatIII sequence – sense strand) which was flanked on both ends with the appropriate (non-converted) primer binding sites and sample barcodes using BBMap v. 38.87[14]. Sample barcodes (Table S8) and both CpGs were degenerated (NNNNNN or NN, respectively). The BAM files were sorted and indexed and taken for further analysis in R v. 4.0.1[7]. Read counts are given in Table S9. The aligned subsequences at the four degenerated positions were extracted from the BAM files using the GenomicAlignments package of the Bioconductor suite[15] and the barcodes demultiplexed using DNABarcodes package[16] using the sequence-Levenstein model for error correction, i.e., allowing insertions and deletions. An alignment was only kept when the barcodes identified the respective sample with high certitude: When both barcodes corresponded without error to the designed barcodes, if one of them was missing, but the other corresponded without error, or when both of them matched the same designed (and unambiguous) barcode within less than four operations. As a last requirement, only those alignments were taken into considerations, in which both CpGs read as “TG” or “CG”. Then, the fraction of “CG” alignments over all alignments for a CpG was determined by sample (defined by treatment, time point and replicate of the conversion). The mean and standard deviation of the three replicate conversions was determined (Table S10, Figure 3c and S14).

RNA extraction and transcriptome analysis by RNA-seq

For transcriptome analysis, 600,000 HEK293T cells were seeded on 3.5 cm dishes and transfected 24 h later with pcDNMT3a (pJaW2559) and pStH1169 in the presence of 0.05 mM DMNB-Cys. After 24h, light irradiation was done as described before and cells were sorted for GFP signal either immediately (0h sample), 4h or 8h later. RNA extraction with TRIzol, RNA-sequencing and subsequent analysis was done as described by Palei et. al.[17]

In short, cleaned, single-end RNA-sequencing reads from BGI Genomics (DNBseq RNA-Seq) were aligned to the GENCODE reference annotation[18] of the human transcriptome (v32) expanded to include transcripts from the transfected plasmids pcDNMT3a and the synthetase/tRNA pair and using the GRCh38 primary assembly (v32) as a decoy. Mapping was performed with Salmon v. 1.3.0[19] adopting a k-mer length of 27 and the --validate Mappings (‘selective alignment’) option[20]. Transcript abundance was quantitated with the same tool considering a fragment length of 160—250 bp (205 ± 45 bp) and correcting for random hexamer priming bias[21] Count data was analyzed with R v. 4.0.1[7] after projecting transcript counts onto genes[22] using DESeq2[23] and applying adaptive shrinkage[24,25] to control the false-discovery rate in differential gene expression.

Western Blot

HEK293T cells were transfected with pcDNMT3a3L/pStH1169 in the presence or absence of DMNB-Cys and if needed light irradiated as described before. 24h after transfection or after light irradiation, cells were harvested by trypsinization, pelleted and lysed with RIPA buffer (Thermo Scientific™ #89900) according to the manufactures protocol. Whole cell lysate was applied to a 10 % SDS-PAGE gel and transferred to a polyvinylidene difluoride (PVDF) membrane using Thermo Scientific Pierce™ Power Blotter. After blocking with LI-COR Odyssey Blocking Buffer and washing with TBS-T buffer, the membrane was incubated with HA-antibody (Cell Signaling #3724) and GAPDH-antibody (Cell Signaling #2118) at 4 °C overnight. After three washing steps with TBS-T, the membrane was incubated with anti-rabbit secondary antibody conjugated with DyLight™ 800 (Cell Signaling #5151), followed by further washing steps and imaging with LI-COR Odyssey® CLx.

Mass spectrometry

The same lysate used for Western Blot was applied to a 10 % SDS-PAGE gel and the part covering the transfection control band was cut out and washed in 200 µL 25 mM NH_4HCO_3 3:1 in Acetonitrile for 30 min 37 °C 600 rpm. Then it was washed in 200 µL 25 mM NH_4HCO_3 1:1 in Acetonitrile for 15 min 37 °C 600 rpm. Both washing steps were repeated once. After reduction with 50 mM DTT in 25 mM NH_4HCO_3 for 45 min 37 °C 600 rpm, the peptides were alkylated in the dark with 55 mM iodacetamide in 25 mM NH_4HCO_3 for 1 h at 25 °C. After two washing steps with 25 mM NH_4HCO_3 1:1 in Acetonitrile, the gel was dehydrated with 100 µL acetonitrile and fully dried afterwards. 125 µL digest solution (0.1 µg/µl Trypsin (in 10 mM HCl) 1:10 in 25 mM NH_4HCO_3) was added and after 15 min at 25 °C another 125 µL 25 mM NH_4HCO_3 was added and shaken at 350 rpm 30 °C overnight. Digestion was stopped by adding 16.6 µL 10 % TFA and samples were sonified on ice for 30 min. The supernatant was saved and twice another 100 µL acetonitrile were added to dry the gel and supernatants were combined and concentrated. After tryptic digestion and purification, the protein fragments were analyzed by nano-HPLC-MS/MS using an UltimateTM 3000 RSLC nano-HPLC system, and a Q ExactiveTM Plus Hybrid Quadrupole-Orbitrap equipped with a nano-spray source (all from ThermoFisher Scientific). Briefly, the lyophilized tryptic peptides were suspended in 20 µL 0.1% TFA and 1 µL of the samples were injected onto and enriched on a C18 PepMap 100 column (5 µm, 100 Å, 300 µm ID * 5 mm, ThermoScientific) using 0.1% TFA, at a flow rate of 30 µL/min, for 5 min. Subsequently, the peptides were separated on a C18 PepMap 100 column (3 µm, 100 Å, 75 µm ID * 50 cm) using a linear gradient, starting with 95% solvent A/5% solvent B and increasing to 30.0% solvent B in 90 min with a flow rate of 300 nL/min (solvent A: water containing 0.1% formic acid; solvent B: acetonitrile containing 0.1% formic acid). The nano-HPLC apparatus was coupled online with the mass spectrometer using a standard coated Pico Tip emitter (ID 20, Tip-ID 10, New Objective, Woburn, MA, USA). Signals in the mass range of m/z 300 to 1650 were acquired at a resolution of 70,000 followed by up to ten high-energy collision-dissociation (HCD) MS/MS scans of the most intense at least doubly charged ions at a resolution of 17,500.

Identification of the artificial modification of the protein of interest was performed by using MaxQuant[26] v.1.6.14.0, including the Andromeda search algorithm and searching a database containing the sequence of the proteins of interest and known contaminants in parallel. Briefly, an MS/MS ion search was performed for enzymatic trypsin cleavage, allowing two missed cleavages. Carbamidomethylation of cysteine, DMNB-modification of cysteine (Ambercodon photoactivated cysteine, formula change C9NO4H9, $\Delta m = 195.05316$), acetylation of protein N-termini, and oxidation of methionine were set as variable modifications. The mass

accuracy was set to 20 ppm for the first search, and to 4.5 ppm for the second search. The false discovery rates for peptide and protein identification were set to 0.01.

Synthesis of 1 (4,5-Dimethoxy-2-Nitrobenzyl-L-Cysteine)

The photocaged amino acid **1** was synthesized according to a procedure adapted from Pedersen *et. al.*[27]
L-Cysteine (2,01 g, 12,75 mmol) was resolved in 1 M NaOH (16 mL), 4,5-Dimethoxy-2-nitrobenzyl bromide resolved in THF (8 mL). The bromide (3,5 g, 12,54 mmol) was added to the stirred L-cysteine solution over 1 h. THF (12 mL) and 1 M NaOH (12 mL) was added and the mixture was stirred overnight. The suspension was filtered and the brown solid was washed with THF, ethyl acetate and Et_2O, dried over filter paper. The crude product was resolved in 20 mL H_2O and lyophilized over 3 days. The product was obtained as a light yellow solid (2,5 g, 7,9 mmol, 61,9 %).

^{1}H NMR (400 MHz, DMSO) δ = 7.67 (s, 1H), 7.32 (s, 1H), 4.08 (dd, *J*=30.1, 13.5, 2H), 3.95 (s, 3H), 3.87 – 3.82 (m, 3H), 2.90 (dd, *J*=14.6, 3.9, 1H), 2.75 (dd, *J*=14.6, 7.5, 1H).

^{13}C NMR (126 MHz, DMSO) δ 169.0, 152.7, 147.4, 139.6, 129.1, 114.9, 108.9, 56.5, 56.1, 53. 3, 40.0, 39.8, 39.7, 39.5, 39.3, 39.2, 39.0, 32.3, 31.8.

SUPPLEMENTARY TABLES

Table S2. Primary Antibodies.

Epitope	Description	Company (Cat. #)
HA	HA-Tag (C29F4) Rabbit mAb	Cell Signaling Technologies (3724)
5mC	Anti-5-methylcytosine Clone 33D3 (mouse monoclonal)	Merck (MABE146)
HSF1	HSF1 Antibody (Rabbit)	Cell Signaling Technology (4356S)

Table S3. Secondary Antibodies.

Description	Company (Cat. #)
Alexa Fluor 405 goat anti-mouse IgG (H+L)	Invitrogen (A31553)
Alexa Fluor 405 goat anti-rabbit IgG (H+L)	Invitrogen (A31556)
Alexa Fluor 488 goat anti-mouse IgG (H+L)	Invitrogen (A11029)
Fluorescein goat anti-rabbit IgG (H+L)	Invitrogen (F2765)
Cyanine5 goat anti-rabbit IgG (H+L)	Invitrogen (A10523)

Table S4. Oligonucleotides for cloning.

All oligonucleotides were synthesized by Sigma-Aldrich.

Name	Sequence (5' → 3')
o2038	CCCTTCTTCTGGCTCTTTGCCAATGTGGTGGCCATGGGCG
o2039	CCATGGCCACCACATTGGCAAAGAGCCAGAAGAAGGGGCG
o2071	AAACGCAAAGTTGGGCGCGCCAACCATGACCAGGAATTTGACC
o2072	GAACGTCGTACGGGTAGTTAATAAGAGGAAGTGAGTTTTGAG
o2175	GATTGGAGGCAGTCCCTAGAATGACCTCTCCATTGTCAACC
o2176	GACAATGGAGAGGTCATTCTAGGGACTGCCTCCAATCACC
o2253	GGCTAGCGCCATGGCTAGCCCGAAAAAGAAACGCAAAGTTG
o2254	CTTTTTCGGGCTAGCCATGGCGCTAGCCAGCTTGGGTCTCC
o2330	ACCGATTCCACCATCCGGGCGGTGTTTCGTCCTTTCCAC
o2331	GGGCGGTATTGCTCTTCCATGGTGGCAAGCTTCCGTGCAG
o2332	CTGCACGGAAGCTTGCCACCATGGAAGAGCAATACCGC
o2333	GTCGAGGCTGATCAGCGGGTTTAGCCAACGACCAGATTG
o2334	CAATCTGGTCGTTGGCTAAACCCGCTGATCAGCCTCGAC
o2335	CAAGTCCCGCTCCGGGTACCATTTTTCGGGGAAATGTGCGC
o2336	GCCCGGATGGTGGAATCGGTAGACACAAGGGATTCTAAATCCCTCGGCGTTCGCGCTGT GCGGGTTCAAGTCCCGCTCCGGGTACCA
o2337	TGGTACCCGGAGCGGGACTTGAACCCGCACAGCGCGAACGCCGAGGGATTTAGAATCC CTTGTGTCTACCGATTCCACCATCCGGGC
o2433	ACAGCAAGGGGGAGGATTGGGAAGACAATAGCAGGCATGC
o2434	CCAATCCTCCCCCTTGCTGTCCTGCCCCACCCCACCCCCCAGAATAGAAT
o2435	ATTAATGTGAGTTAGCTCACTCATTAGGCACCCCAGGCTT
o2436	AGTGAGCTAACTCACATTAATTGCGTTGCGCTCACTGCCC
o2475	CTACCCGCCCGGACGCCTTTATGGGTTGTAC
o2476	ACAACCCATAAAGGCGTCCGGGCGGGTAGTG
o3352	CTGACGTCGACGGATCGGGAGCGGCCGCTTCGAGCAGACA
o3355	TGTCTGCTCGAAGCGGCCGCTCCCGATCCGTCGACGTCAG
o3569	GGAATATTTTGCTTGTGTGATTAACTACCCGTACGACGTTC
o3570	GAACGTCGTACGGGTAGTTAATCACACAAGCAAAATATTCC
o3574	ATGGTAAGCCCTCCCGTATCGTAGTTATCTACACGACGGG
o3575	ACTACGATACGGGAGGGCTTACCATCTGGCCCCAGTGCTG
o3672	GCAGTCCCTagAATGACCTCTgCATTGTCAACCCTGCCCG
o3673	CGGGCAGGGTTGACAATGcAGAGGTCATTctAGGGACTGC
o3674	GCCTCTTCTTTGAGTTCTACCaCCTCCTGCATGATGCGCG
o3675	CGCGCATCATGCAGGAGGtGGTAGAACTCAAAGAAGAGGC
o3676	GTGACAAGAGGGACATCTCGCaATTTCTTGAGTCTAACCCC
o3677	GGGGTTAGACTCAAGAAATtGCGAGATGTCCCTCTTGTCAC

Name	Sequence (5’ → 3’)
o3678	CAAGAGGGACATCTCGgGATTTCTTGAGTCTAACCCCGTG
o3679	CACGGGGTTAGACTCAAGAAATCcCGAGATGTCCCTCTTG
o3680	GCAAAGTGAGGACCATTACCAtgAGGTCAAACTCTATAAAG
o3681	CTTTATAGAGTTTGACCTcaTGGTAATGGTCCTCACTTTGC
o3684	GTGAGGACCATTACCACCgccTCAAACTCTATAAAGCAG
o3685	CTGCTTTATAGAGTTTGAggcGGTGGTAATGGTCCTCAC
o3686	GACGTCTCCAACATGAGCtGCTTGGCGAGGCAGAGACTGCT
o3687	AGCAGTCTCTGCCTCGCCAAGCaGCTCATGTTGGAGACGTC
o3688	GACGTCTCCAACATGAGCCaCTTGGCGAGGCAGAGACTGCT
o3689	AGCAGTCTCTGCCTCGCCAAGtGGCTCATGTTGGAGACGTC
o3690	CTGCTGGGCCGATCGTcGAGCGTGCCGGTCATCCGCCACC
o3691	GGTGGCGGATGACCGGCACGCTCgACGATCGGCCCAGCAG

Table S5. Oligonucleotides for bisulfite PCR.

Name	Sequence (5’ → 3’)
o3266_fw SatIII Pyroseq.	GGAATGGATTTAATTTGAATG
o3439_rv SatIII Pyroseq.	[Btn]TTCCATTCCATTCCTATACT
o3268_fw SatIII Sanger	CTTCCTGGCACGAGGGAATGGATTTAATTTGAATG
o3269_rv SatIII Sanger	GAAACAGCTATGACTTCCATTCCATTCCTATACT
o2348_fw BRCA1 Sanger	CTTCCTGGCACGAGATTGGGTGGTTAATTTAGAG
o2350_fw BRCA1 Sanger	CTTCCTGGCACGAGGGGAATTATAGATAAATTAA
o2220_rv BRCA1 Sanger	CCAATACCCCAAAACATCAC
o2339_fw p16_1	CTTCCTGGCACGAGGGGTAGGTGGGGAGGAGTTTAG
o2199_rv p16_1	ATATCTTTCCAAACAAAAAAAC
o2340_fw p16_2	CTTCCTGGCACGAGGGGGGAGATTTAATTTGGGG
o2201_rv p16_2	AACCCCTCCTCTTTCTTCCTCC
o2344_fw p16 Sanger	CTTCCTGGCACGAGGGTGGGGAGGAGTTTAGT
o2800_rv p16 Sanger	CTATCCCTCAAATCCTC
o2363	ATCACGCTTCCTGGCACGAG
o2364	CGATGTCTTCCTGGCACGAG
o2365	TTAGGCCTTCCTGGCACGAG
o2366	TGACCACTTCCTGGCACGAG
o2367	ACAGTGCTTCCTGGCACGAG
o2368	GCCAATCTTCCTGGCACGAG
o2369	CAGATCCTTCCTGGCACGAG
o2370	ACTTGACTTCCTGGCACGAG
o2371	GATCAGCTTCCTGGCACGAG
o2372	TAGCTTCTTCCTGGCACGAG
o2373	GGCTACCTTCCTGGCACGAG
o2374	CTTGTACTTCCTGGCACGAG
o2375	AGTCAACTTCCTGGCACGAG
o2376	AGTTCCCTTCCTGGCACGAG
o2377	ATGTCACTTCCTGGCACGAG
o2378	CCGTCCCTTCCTGGCACGAG
o2379	GTAGAGCTTCCTGGCACGAG
o2380	GTCCGCCTTCCTGGCACGAG
o2381	GTGAAACTTCCTGGCACGAG
o2382	GTGGCCCTTCCTGGCACGAG
o2383	GTTTCGCTTCCTGGCACGAG
o2384	CGTACGCTTCCTGGCACGAG
o2385	GAGTGGCTTCCTGGCACGAG
o2386	GGTAGCCTTCCTGGCACGAG
o2387	ACTGATCTTCCTGGCACGAG
o2388	ATGAGCCTTCCTGGCACGAG
o2389	ATTCCTCTTCCTGGCACGAG
o2390	CAAAAGCTTCCTGGCACGAG
o2391	CAACTACTTCCTGGCACGAG

Name	Sequence (5’ → 3’)
o2392	CACCGGCTTCCTGGCACGAG
o2399	CGGAATCTTCCTGGCACGAG
o2400	CTAGCTCTTCCTGGCACGAG
o2401	CTATACCTTCCTGGCACGAG
o3033	ATCACGGAAACAGCTATGAC
o3034	CGATGTGAAACAGCTATGAC
o3035	TTAGGCGAAACAGCTATGAC
o3036	TGACCAGAAACAGCTATGAC
o3037	ACAGTGGAAACAGCTATGAC
o3038	GCCAATGAAACAGCTATGAC
o3039	CAGATCGAAACAGCTATGAC
o3040	ACTTGAGAAACAGCTATGAC
o3041	GATCAGGAAACAGCTATGAC
o3042	TAGCTTGAAACAGCTATGAC
o3043	GGCTACGAAACAGCTATGAC
o3044	CTTGTAGAAACAGCTATGAC
o3045	AGTCAAGAAACAGCTATGAC
o3046	AGTTCCGAAACAGCTATGAC
o3047	ATGTCAGAAACAGCTATGAC
o3048	CCGTCCGAAACAGCTATGAC
o3049	GTAGAGGAAACAGCTATGAC
o3050	GTCCGCGAAACAGCTATGAC
o3051	GTGAAAGAAACAGCTATGAC
o3052	GTGGCCGAAACAGCTATGAC
o3053	GTTTCGGAAACAGCTATGAC
o3054	CGTACGGAAACAGCTATGAC
o3055	GAGTGGGAAACAGCTATGAC
o3056	GGTAGCGAAACAGCTATGAC
o3057	ACTGATGAAACAGCTATGAC
o3058	ATGAGCGAAACAGCTATGAC
o3059	ATTCCTGAAACAGCTATGAC
o3060	CAAAAGGAAACAGCTATGAC
o3061	CAACTAGAAACAGCTATGAC
o3062	CACCGGGAAACAGCTATGAC
o3069	CGGAATGAAACAGCTATGAC
o3070	CTAGCTGAAACAGCTATGAC
o3071	CTATACGAAACAGCTATGAC

Table S6. Oligonucleotides for RT-qPCR.

Name	Sequence (5’ → 3’)
o3835_fw ARC	GCAGGGCTCCGTGAAGAAC
o3836_rv ARC	CGTCCACGTAGAGCGTCTGG
o3845_fw GAPDH	AAGGTGAAGGTCGGAGTCAA
o3846_rv GAPDH	GGAAGATGGTGATGGGATTT
o4138_fw PTX3	CGAAATAGACAATGGACTCCATCC [28]
o4139_rv PTX3	GCAGGCGCACGGCGT [28]

Table S7. Mutation table of COSMIC database with frequencies.

Mutation	COSMIC Database Count	Genomic Mutation ID
S714C	25	COSV53039274
R736H	17	COSV53036512
R771Q	8	COSV53037113
R771G	1	COSV53073691
T835M	5	COSV53041391
R836A	0	-
R882C	358	COSV53036332
R882H	858	COSV53036153
W893S	12	COSV53041651

Table S8. Sample assignments for SatIII bisulfite-converted amplicons.

Run ID	Treatment	Time Point	Replicate	Primer fw	Primer rv	Barcode fw
Sample1	with_light	0	Rep1	o2363	o3033	ATCACG
Sample1	with_light	0	Rep2	o2364	o3034	CGATGT
Sample1	with_light	0	Rep3	o2365	o3035	TTAGGC
Sample1	no_light	0	Rep1	o2366	o3036	TGACCA
Sample1	no_light	0	Rep2	o2367	o3037	ACAGTG
Sample1	no_light	0	Rep3	o2368	o3038	GCCAAT
Sample1	with_light	2	Rep1	o2369	o3039	CAGATC
Sample1	with_light	2	Rep2	o2370	o3040	ACTTGA
Sample1	with_light	2	Rep3	o2371	o3041	GATCAG
Sample1	no_light	2	Rep1	o2372	o3042	TAGCTT
Sample1	no_light	2	Rep2	o2373	o3043	GGCTAC
Sample1	no_light	2	Rep3	o2374	o3044	CTTGTA
Sample1	with_light	6	Rep1	o2375	o3045	AGTCAA
Sample1	with_light	6	Rep2	o2376	o3046	AGTTCC
Sample1	with_light	6	Rep3	o2377	o3047	ATGTCA
Sample1	no_light	6	Rep1	o2378	o3048	CCGTCC
Sample1	no_light	6	Rep2	o2379	o3049	GTAGAG
Sample1	no_light	6	Rep3	o2380	o3050	GTCCGC
Sample1	with_light	10	Rep1	o2381	o3051	GTGAAA
Sample2	with_light	10	Rep2	o2382	o3052	GTGGCC
Sample2	with_light	10	Rep3	o2383	o3053	GTTTCG
Sample2	no_light	10	Rep1	o2384	o3054	CGTACG
Sample2	no_light	10	Rep2	o2385	o3055	GAGTGG
Sample2	no_light	10	Rep3	o2386	o3056	GGTAGC
Sample2	with_light	24	Rep1	o2387	o3057	ACTGAT
Sample2	with_light	24	Rep2	o2388	o3058	ATGAGC
Sample2	with_light	24	Rep3	o2389	o3059	ATTCCT
Sample2	no_light	24	Rep1	o2390	o3060	CAAAAG
Sample2	no_light	24	Rep2	o2391	o3061	CAACTA
Sample2	no_light	24	Rep3	o2392	o3062	CACCGG
Sample2	untransfected	24	Rep1	o2399	o3069	CGGAAT
Sample2	untransfected	24	Rep2	o2400	o3070	CTAGCT
Sample2	untransfected	24	Rep3	o2401	o3071	CTATAC

Table S9. Read counts of SatIII bisulfite-converted amplicons by analysis stage.

Run ID	Raw Reads	Merged Reads	Mapped Reads	Assigned after Demultiplexing	CpG1 AND CpG2
Sample1	230,677	227,214 (99%)	219,207 (96%)	134,685 (61%)	114,296 (85%)
Sample2	190,166	185,797 (97%)	175,217 (95%)	120,999 (69%)	99,530 (82%)

Table S10. Methylation levels of both SatIII CpGs.

Time Point	Treatment	Replicate	Name	CG	TG	5mC [%]	Reads
0	Without Light	Rep1	First CpG	137	3898	3,40	4035
0	Without Light	Rep1	Second CpG	338	3697	8,38	4035
0	Without Light	Rep2	First CpG	288	6785	4,07	7073
0	Without Light	Rep2	Second CpG	483	6590	6,83	7073
0	Without Light	Rep3	First CpG	314	7236	4,16	7550
0	Without Light	Rep3	Second CpG	557	6993	7,38	7550
0	With Light	Rep1	First CpG	353	8555	3,96	8908
0	With Light	Rep1	Second CpG	802	8106	9,00	8908
0	With Light	Rep2	First CpG	172	3930	4,19	4102
0	With Light	Rep2	Second CpG	335	3767	8,17	4102
0	With Light	Rep3	First CpG	175	3060	5,41	3235
0	With Light	Rep3	Second CpG	136	3099	4,20	3235
2	Without Light	Rep1	First CpG	158	3096	4,86	3254
2	Without Light	Rep1	Second CpG	205	3049	6,30	3254
2	Without Light	Rep2	First CpG	159	3068	4,93	3227
2	Without Light	Rep2	Second CpG	267	2960	8,27	3227
2	Without Light	Rep3	First CpG	229	5275	4,16	5504
2	Without Light	Rep3	Second CpG	402	5102	7,30	5504
2	With Light	Rep1	First CpG	348	5195	6,28	5543
2	With Light	Rep1	Second CpG	384	5159	6,93	5543
2	With Light	Rep2	First CpG	341	4607	6,89	4948
2	With Light	Rep2	Second CpG	466	4482	9,42	4948
2	With Light	Rep3	First CpG	493	5927	7,68	6420
2	With Light	Rep3	Second CpG	462	5958	7,20	6420
6	Without Light	Rep1	First CpG	123	2650	4,44	2773
6	Without Light	Rep1	Second CpG	224	2549	8,08	2773
6	Without Light	Rep2	First CpG	371	8023	4,42	8394
6	Without Light	Rep2	Second CpG	577	7817	6,87	8394
6	Without Light	Rep3	First CpG	291	7371	3,80	7662
6	Without Light	Rep3	Second CpG	602	7060	7,86	7662
6	With Light	Rep1	First CpG	831	7764	9,67	8595
6	With Light	Rep1	Second CpG	653	7942	7,60	8595
6	With Light	Rep2	First CpG	564	6262	8,26	6826
6	With Light	Rep2	Second CpG	550	6276	8,06	6826
6	With Light	Rep3	First CpG	880	6948	11,24	7828
6	With Light	Rep3	Second CpG	891	6937	11,38	7828
10	Without Light	Rep1	First CpG	213	1983	9,70	2196
10	Without Light	Rep1	Second CpG	184	2012	8,38	2196
10	Without Light	Rep2	First CpG	237	4612	4,89	4849
10	Without Light	Rep2	Second CpG	349	4500	7,20	4849
10	Without Light	Rep3	First CpG	192	4154	4,42	4346
10	Without Light	Rep3	Second CpG	331	4015	7,62	4346
10	With Light	Rep1	First CpG	1087	7331	12,91	8418
10	With Light	Rep1	Second CpG	834	7584	9,91	8418
10	With Light	Rep2	First CpG	688	4968	12,16	5656
10	With Light	Rep2	Second CpG	612	5044	10,82	5656
10	With Light	Rep3	First CpG	1627	5993	21,35	7620
10	With Light	Rep3	Second CpG	798	6822	10,47	7620
24	Without Light	Rep1	First CpG	207	3796	5,17	4003
24	Without Light	Rep1	Second CpG	332	3671	8,29	4003
24	Without Light	Rep2	First CpG	207	3507	5,57	3714
24	Without Light	Rep2	Second CpG	233	3481	6,27	3714
24	Without Light	Rep3	First CpG	163	2613	5,87	2776
24	Without Light	Rep3	Second CpG	214	2562	7,71	2776
24	Untransfected + Light	Rep1	First CpG	141	4664	2,93	4805
24	Untransfected + Light	Rep1	Second CpG	455	4350	9,47	4805

Time Point	Treatment	Replicate	Name	CG	TG	5mC [%]	Reads
24	Untransfected + Light	Rep2	First CpG	318	5645	5,33	5963
24	Untransfected + Light	Rep2	Second CpG	499	5464	8,37	5963
24	Untransfected + Light	Rep3	First CpG	262	7271	3,48	7533
24	Untransfected + Light	Rep3	Second CpG	674	6859	8,95	7533
24	With Light	Rep1	First CpG	1232	5850	17,40	7082
24	With Light	Rep1	Second CpG	844	6238	11,92	7082
24	With Light	Rep2	First CpG	1446	6987	17,15	8433
24	With Light	Rep2	Second CpG	1054	7379	12,50	8433
24	With Light	Rep3	First CpG	1275	4653	21,51	5928
24	With Light	Rep3	Second CpG	818	5110	13,80	5928

Table S11. Description of RNA sequencing samples and mapping statistics. Alignment was done with Salmon v. 1.3.0 against an expanded version of the human transcriptome including transcripts originating from the transfected plasmids.

Run ID	Light	Time	Clean Reads	Mapping Rate	Mapped Counts [1e6]
V300055926_L2_HK500HUMhliEAAARAAPEI-509_1	yes	0h	36281971	90.37%	32.8
V300055926_L2_HK500HUMhliEAABRAAPEI-510_1	yes	0h	35360906	90.72%	32.1
V300055926_L2_HK500HUMhliEAACRAAPEI-511_1	yes	4h	35204944	89.92%	31.7
V300055926_L2_HK500HUMhliEAADRAAPEI-512_1	yes	4h	35348904	90.47%	32.0
V300055926_L2_HK500HUMhliEAAERAAPEI-513_1	yes	8h	43547913	89.78%	39.1
V300055926_L2_HK500HUMhliEAAFRAAPEI-514_1	yes	8h	41683354	90.93%	37.9
V300055926_L2_HK500HUMhliEAAIRAAPEI-517_1	no	0h	37410535	89.77%	33.6
V300055926_L2_HK500HUMhliEAAJRAAPEI-518_1	no	0h	36268131	89.88%	32.6
V300055926_L2_HK500HUMhliEAAKRAAPEI-519_1	no	4h	34718412	90.32%	31.4
V300055926_L2_HK500HUMhliEAALRAAPEI-520_1	no	4h	35230629	90.7%	32.0
V300055912_L3_HK500HUMhliEAAMRAAPEI-521_1	no	8h	34629709	89.84%	31.1
V300055912_L3_HK500HUMhliEAANRAAPEI-522_1	no	8h	36161999	89.21%	32.3

Table S12. Number of genes reported differentially expressed upon light-induction after 4h and 8h. Absolute log2-fold change (LFC) and significance level of 0.05. Percentage reported with respect to 39,520 genes that had non-zero counts.

Time	LFC	Up-Regulated	Down-Regulated
4h	>0	68 (0.17%)	30 (0.08%)
8h	>0	19 (0.05%)	79 (0.20%)

Table S13. Log2-fold changes of genes after 4h.

ENSEMBL	Symbol	BaseMean	Log2FoldChange	lfcSE	Stat	pvalue	padj
ENSG00000257225.1	–	14.13	-14.33	2.61	38.02	5.55E-09	1.99E-05
ENSG00000187664.9	HAPLN4	10.79	-13.40	2.73	24.03	6.04E-06	4.50E-03
ENSG00000259607.1	–	8.87	-8.78	2.18	16.97	2.06E-04	4.61E-02
ENSG00000275496.4	LOC102724701	48.52	-8.16	1.65	42.37	6.31E-10	3.77E-06
ENSG00000198237.8	–	21.42	-5.75	2.02	18.78	8.38E-05	2.54E-02

ENSEMBL	Symbol	BaseMean	Log2FoldChange	lfcSE	Stat	pvalue	padj
ENSG00000277399.4	GPR179	20.53	-5.70	1.62	16.69	2.37E-04	4.76E-02
ENSG00000196218.12	RYR1	44.14	-3.09	1.14	17.00	2.03E-04	4.61E-02
ENSG00000256591.5	–	73.85	-2.81	0.76	17.69	1.44E-04	3.58E-02
ENSG00000142178.9	SIK1	78.82	-1.47	1.20	19.10	7.11E-05	2.32E-02
ENSG00000130635.15	COL5A1	290.37	-1.22	0.35	27.92	8.64E-07	1.19E-03
ENSG00000143499.14	SMYD2	292.08	-1.18	0.35	17.05	1.98E-04	4.61E-02
ENSG00000187122.17	SLIT1	210.61	-1.10	0.48	24.54	4.69E-06	4.00E-03
ENSG00000135709.12	KIAA0513	269.09	-0.96	0.41	18.27	1.08E-04	3.02E-02
ENSG00000283154.2	SCHIP1	201.62	-0.77	0.34	19.94	4.67E-05	1.86E-02
ENSG00000155254.13	MARVELD1	500.88	-0.77	0.36	19.07	7.24E-05	2.32E-02
ENSG00000130940.15	CASZ1	581.09	-0.66	0.23	36.03	1.50E-08	4.48E-05
ENSG00000174705.13	SH3PXD2B	1138.35	-0.62	0.27	40.15	1.91E-09	8.55E-06
ENSG00000119446.14	RBM18	796.72	-0.50	0.27	34.68	2.95E-08	7.56E-05
ENSG00000076043.10	REXO2	428.79	-0.49	0.25	16.57	2.52E-04	4.80E-02
ENSG00000118197.14	DDX59	146.59	-0.44	0.41	16.48	2.63E-04	4.92E-02
ENSG00000109911.19	ELP4	554.11	-0.41	0.28	16.64	2.43E-04	4.76E-02
ENSG00000165533.18	TTC8	412.62	-0.28	0.42	16.48	2.64E-04	4.92E-02
ENSG00000155097.12	ATP6V1C1	475.48	-0.24	0.41	17.48	1.60E-04	3.92E-02
ENSG00000136156.15	ITM2B	1743.03	-0.24	0.22	20.03	4.47E-05	1.86E-02
ENSG00000104549.12	SQLE	741.09	-0.23	0.31	20.82	3.01E-05	1.50E-02
ENSG00000043514.17	TRIT1	1188.44	-0.14	0.24	16.86	2.19E-04	4.61E-02
ENSG00000133835.16	HSD17B4	970.84	-0.13	0.20	18.95	7.68E-05	2.37E-02
ENSG00000113558.18	SKP1	777.43	-0.05	0.23	21.21	2.48E-05	1.39E-02
ENSG00000152942.19	RAD17	448.81	-0.03	0.34	19.49	5.87E-05	2.19E-02
ENSG00000186472.20	PCLO	219.03	-0.01	0.70	29.55	3.84E-07	7.64E-04
ENSG00000100554.12	ATP6V1D	456.45	0.02	0.26	18.74	8.53E-05	2.55E-02
ENSG00000164294.14	GPX8	281.17	0.03	0.39	24.19	5.58E-06	4.50E-03
ENSG00000117528.14	ABCD3	727.67	0.04	0.31	21.07	2.66E-05	1.40E-02
ENSG00000139973.16	SYT16	34.02	0.05	0.91	24.97	3.78E-06	3.98E-03
ENSG00000116489.13	CAPZA1	2492.82	0.13	0.25	17.73	1.42E-04	3.57E-02
ENSG00000137040.10	RANBP6	208.85	0.15	0.44	16.63	2.45E-04	4.76E-02
ENSG00000229018.5	–	18.01	0.17	0.96	17.90	1.30E-04	3.40E-02
ENSG00000144840.9	RABL3	210.33	0.19	0.51	16.98	2.05E-04	4.61E-02
ENSG00000102901.13	CENPT	1916.58	0.20	0.22	16.92	2.12E-04	4.61E-02
ENSG00000213186.8	TRIM59	259.19	0.23	0.42	17.72	1.42E-04	3.57E-02
ENSG00000124333.16	VAMP7	1223.52	0.24	0.25	18.22	1.11E-04	3.02E-02
ENSG00000115540.15	MOB4	559.47	0.30	0.27	24.60	4.56E-06	4.00E-03
ENSG00000008710.19	PKD1	1529.40	0.31	0.23	17.28	1.77E-04	4.28E-02
ENSG00000151690.15	MFSD6	58.86	0.33	0.64	19.98	4.59E-05	1.86E-02
ENSG00000117569.18	PTBP2	1576.88	0.33	0.32	24.68	4.37E-06	4.00E-03
ENSG00000137575.12	SDCBP	616.76	0.35	0.26	23.94	6.33E-06	4.50E-03
ENSG00000065615.14	CYB5R4	205.46	0.36	0.43	16.44	2.69E-04	4.92E-02
ENSG00000164023.14	SGMS2	201.14	0.37	0.41	21.36	2.30E-05	1.37E-02
ENSG00000153914.16	SREK1	694.96	0.39	0.40	16.91	2.13E-04	4.61E-02
ENSG00000139218.18	SCAF11	604.61	0.41	0.40	16.44	2.69E-04	4.92E-02
ENSG00000213639.10	PPP1CB	1483.96	0.42	0.27	21.07	2.66E-05	1.40E-02
ENSG00000107290.14	SETX	611.62	0.44	0.34	20.52	3.49E-05	1.62E-02
ENSG00000135535.17	CD164	1101.65	0.45	0.26	23.69	7.18E-06	4.77E-03
ENSG00000211456.12	SACM1L	347.14	0.45	0.36	19.35	6.28E-05	2.30E-02
ENSG00000084073.9	ZMPSTE24	1386.77	0.46	0.25	21.20	2.49E-05	1.39E-02
ENSG00000143952.20	VPS54	374.66	0.49	0.37	18.65	8.90E-05	2.61E-02
ENSG00000111530.13	CAND1	1560.57	0.50	0.29	16.79	2.26E-04	4.70E-02
ENSG00000172795.16	DCP2	508.89	0.50	0.38	19.19	6.82E-05	2.32E-02
ENSG00000123505.17	AMD1	2613.63	0.50	0.24	28.87	5.39E-07	8.78E-04
ENSG00000111860.14	CEP85L	359.31	0.51	0.41	16.70	2.36E-04	4.76E-02
ENSG00000118496.5	FBXO30	279.56	0.52	0.49	16.98	2.06E-04	4.61E-02
ENSG00000047410.14	TPR	1007.40	0.53	0.39	20.22	4.06E-05	1.78E-02
ENSG00000163743.13	RCHY1	503.70	0.56	0.29	20.45	3.63E-05	1.62E-02
ENSG00000134987.11	WDR36	707.44	0.56	0.38	16.85	2.19E-04	4.61E-02
ENSG00000260317.1	–	56.91	0.56	0.67	25.32	3.18E-06	3.56E-03
ENSG00000254535.4	PABPC4L	262.88	0.59	0.38	18.28	1.07E-04	3.02E-02
ENSG00000197045.13	GMFB	465.59	0.59	0.32	20.48	3.56E-05	1.62E-02
ENSG00000153922.10	CHD1	649.21	0.59	0.47	16.66	2.41E-04	4.76E-02
ENSG00000089154.11	GCN1	5266.75	0.65	0.29	26.36	1.89E-06	2.26E-03
ENSG00000198887.9	SMC5	554.70	0.69	0.45	19.78	5.06E-05	1.97E-02
ENSG00000126217.21	MCF2L	128.46	0.70	0.53	18.22	1.10E-04	3.02E-02
ENSG00000188419.14	CHM	506.00	0.70	0.37	20.57	3.41E-05	1.62E-02
ENSG00000180008.9	SOCS4	390.43	0.70	0.50	19.26	6.56E-05	2.30E-02
ENSG00000119787.14	ATL2	636.20	0.74	0.32	19.00	7.47E-05	2.35E-02
ENSG00000162601.11	MYSM1	1501.36	0.81	0.42	19.07	7.21E-05	2.32E-02
ENSG00000162409.11	PRKAA2	1053.00	0.81	0.39	19.09	7.14E-05	2.32E-02
ENSG00000177565.16	TBL1XR1	623.67	0.84	0.39	16.88	2.16E-04	4.61E-02

ENSEMBL	Symbol	BaseMean	Log2FoldChange	lfcSE	Stat	pvalue	padj
ENSG00000137145.20	DENND4C	393.43	0.89	0.47	17.93	1.28E-04	3.40E-02
ENSG00000198826.11	ARHGAP11A	301.12	0.91	0.47	29.32	4.30E-07	7.70E-04
ENSG00000134318.14	ROCK2	241.38	0.93	0.49	18.21	1.11E-04	3.02E-02
ENSG00000167971.16	CASKIN1	636.11	0.99	0.44	27.43	1.11E-06	1.42E-03
ENSG00000144893.12	MED12L	301.03	0.99	0.51	16.76	2.29E-04	4.72E-02
ENSG00000078177.14	N4BP2	156.39	1.03	0.54	16.96	2.08E-04	4.61E-02
ENSG00000166575.17	TMEM135	270.80	1.05	0.41	22.32	1.42E-05	8.77E-03
ENSG00000023516.9	AKAP11	511.75	1.06	0.45	20.88	2.92E-05	1.49E-02
ENSG00000139618.15	BRCA2	389.39	1.34	0.60	28.14	7.75E-07	1.16E-03
ENSG00000135968.20	GCC2	189.05	1.45	0.44	22.39	1.37E-05	8.77E-03
ENSG00000137573.14	SULF1	41.31	1.50	0.94	19.49	5.87E-05	2.19E-02
ENSG00000261098.1	–	72.00	1.51	0.57	29.81	3.36E-07	7.52E-04
ENSG00000285053.1	TBCE	155.22	2.21	0.83	24.59	4.58E-06	4.00E-03
ENSG00000196628.18	TCF4	240.24	2.52	0.55	24.03	6.04E-06	4.50E-03
ENSG00000188611.15	ASAH2	125.17	2.59	1.01	17.88	1.31E-04	3.40E-02
ENSG00000103426.12	CORO7-PAM16	65.29	2.63	0.75	79.71	4.90E-18	8.78E-14
ENSG00000160219.12	GAB3	64.96	2.86	0.67	20.10	4.32E-05	1.84E-02
ENSG00000151006.7	PRSS53	68.44	2.87	0.65	19.28	6.50E-05	2.30E-02
ENSG00000198064.13	NPIPB13	21.84	5.27	1.82	23.88	6.53E-06	4.50E-03
ENSG00000280987.4	MATR3	124.60	5.66	0.92	72.68	1.65E-16	1.48E-12
ENSG00000261247.1	GOLGA8T	20.36	6.24	1.85	16.61	2.47E-04	4.76E-02

Table S14. Log2-fold changes of genes after 8h.

ENSEMBL	Symbol	BaseMean	Log2FoldChange	lfcSE	Stat	pvalue	padj
ENSG00000257225.1	–	14.13	-13.87	2.61	38.02	5.55E-09	1.99E-05
ENSG00000229018.5	–	18.01	-6.12	1.73	17.90	1.30E-04	3.40E-02
ENSG00000259607.1	–	8.87	-6.05	2.13	16.97	2.06E-04	4.61E-02
ENSG00000198064.13	NPIPB13	21.84	-5.93	2.08	23.88	6.53E-06	4.50E-03
ENSG00000187664.9	HAPLN4	10.79	-5.37	2.35	24.03	6.04E-06	4.50E-03
ENSG00000142178.9	SIK1	78.82	-5.37	1.19	19.10	7.11E-05	2.32E-02
ENSG00000139973.16	SYT16	34.02	-4.94	1.16	24.97	3.78E-06	3.98E-03
ENSG00000196218.12	RYR1	44.14	-4.80	1.13	17.00	2.03E-04	4.61E-02
ENSG00000186472.20	PCLO	219.03	-3.46	0.71	29.55	3.84E-07	7.64E-04
ENSG00000260317.1	–	56.91	-3.09	0.78	25.32	3.18E-06	3.56E-03
ENSG00000137573.14	SULF1	41.31	-2.86	0.98	19.49	5.87E-05	2.19E-02
ENSG00000256591.5	–	73.85	-2.85	0.76	17.69	1.44E-04	3.58E-02
ENSG00000151690.15	MFSD6	58.86	-2.60	0.70	19.98	4.59E-05	1.86E-02
ENSG00000139618.15	BRCA2	389.39	-1.93	0.61	28.14	7.75E-07	1.16E-03
ENSG00000188611.15	ASAH2	125.17	-1.86	1.02	17.88	1.31E-04	3.40E-02
ENSG00000261098.1	–	72.00	-1.83	0.64	29.81	3.36E-07	7.52E-04
ENSG00000144840.9	RABL3	210.33	-1.81	0.53	16.98	2.05E-04	4.61E-02
ENSG00000164294.14	GPX8	281.17	-1.69	0.39	24.19	5.58E-06	4.50E-03
ENSG00000118197.14	DDX59	146.59	-1.68	0.42	16.48	2.63E-04	4.92E-02
ENSG00000198826.11	ARHGAP11A	301.12	-1.67	0.48	29.32	4.30E-07	7.70E-04
ENSG00000165533.18	TTC8	412.62	-1.63	0.42	16.48	2.64E-04	4.92E-02
ENSG00000119446.14	RBM18	796.72	-1.61	0.28	34.68	2.95E-08	7.56E-05
ENSG00000155097.12	ATP6V1C1	475.48	-1.61	0.41	17.48	1.60E-04	3.92E-02
ENSG00000126217.21	MCF2L	128.46	-1.56	0.53	18.22	1.10E-04	3.02E-02
ENSG00000137040.10	RANBP6	208.85	-1.53	0.45	16.63	2.45E-04	4.76E-02
ENSG00000283154.2	SCHIP1	201.62	-1.53	0.34	19.94	4.67E-05	1.86E-02
ENSG00000180008.9	SOCS4	390.43	-1.53	0.51	19.26	6.56E-05	2.30E-02
ENSG00000164023.14	SGMS2	201.14	-1.52	0.43	21.36	2.30E-05	1.37E-02
ENSG00000118496.5	FBXO30	279.56	-1.50	0.50	16.98	2.06E-04	4.61E-02
ENSG00000213186.8	TRIM59	259.19	-1.49	0.44	17.72	1.42E-04	3.57E-02
ENSG00000065615.14	CYB5R4	205.46	-1.36	0.44	16.44	2.69E-04	4.92E-02
ENSG00000104549.12	SQLE	741.09	-1.35	0.31	20.82	3.01E-05	1.50E-02
ENSG00000152942.19	RAD17	448.81	-1.34	0.34	19.49	5.87E-05	2.19E-02
ENSG00000143499.14	SMYD2	292.08	-1.33	0.35	17.05	1.98E-04	4.61E-02
ENSG00000153922.10	CHD1	649.21	-1.32	0.48	16.66	2.41E-04	4.76E-02
ENSG00000198887.9	SMC5	554.70	-1.31	0.45	19.78	5.06E-05	1.97E-02
ENSG00000078177.14	N4BP2	156.39	-1.31	0.57	16.96	2.08E-04	4.61E-02
ENSG00000117528.14	ABCD3	727.67	-1.23	0.31	21.07	2.66E-05	1.40E-02
ENSG00000153914.16	SREK1	694.96	-1.22	0.41	16.91	2.13E-04	4.61E-02
ENSG00000134318.14	ROCK2	241.38	-1.21	0.50	18.21	1.11E-04	3.02E-02
ENSG00000047410.14	TPR	1007.40	-1.19	0.39	20.22	4.06E-05	1.78E-02
ENSG00000117569.18	PTBP2	1576.88	-1.19	0.32	24.68	4.37E-06	4.00E-03
ENSG00000111860.14	CEP85L	359.31	-1.18	0.42	16.70	2.36E-04	4.76E-02
ENSG00000139218.18	SCAF11	604.61	-1.17	0.40	16.44	2.69E-04	4.92E-02
ENSG00000172795.16	DCP2	508.89	-1.16	0.38	19.19	6.82E-05	2.32E-02

ENSEMBL	Symbol	BaseMean	Log2FoldChange	lfcSE	Stat	pvalue	padj
ENSG00000137145.20	DENND4C	393.43	-1.15	0.48	17.93	1.28E-04	3.40E-02
ENSG00000211456.12	SACM1L	347.14	-1.15	0.37	19.35	6.28E-05	2.30E-02
ENSG00000109911.19	ELP4	554.11	-1.14	0.28	16.64	2.43E-04	4.76E-02
ENSG00000144893.12	MED12L	301.03	-1.14	0.52	16.76	2.29E-04	4.72E-02
ENSG00000143952.20	VPS54	374.66	-1.10	0.38	18.65	8.90E-05	2.61E-02
ENSG00000254535.4	PABPC4L	262.88	-1.08	0.39	18.28	1.07E-04	3.02E-02
ENSG00000107290.14	SETX	611.62	-1.07	0.34	20.52	3.49E-05	1.62E-02
ENSG00000162601.11	MYSM1	1501.36	-1.05	0.42	19.07	7.21E-05	2.32E-02
ENSG00000023516.9	AKAP11	511.75	-1.04	0.46	20.88	2.92E-05	1.49E-02
ENSG00000076043.10	REXO2	428.79	-1.03	0.25	16.57	2.52E-04	4.80E-02
ENSG00000134987.11	WDR36	707.44	-1.01	0.39	16.85	2.19E-04	4.61E-02
ENSG00000115540.15	MOB4	559.47	-1.01	0.27	24.60	4.56E-06	4.00E-03
ENSG00000100554.12	ATP6V1D	456.45	-0.99	0.27	18.74	8.53E-05	2.55E-02
ENSG00000188419.14	CHM	506.00	-0.99	0.37	20.57	3.41E-05	1.62E-02
ENSG00000113558.18	SKP1	777.43	-0.97	0.24	21.21	2.48E-05	1.39E-02
ENSG00000136156.15	ITM2B	1743.03	-0.96	0.22	20.03	4.47E-05	1.86E-02
ENSG00000162409.11	PRKAA2	1053.00	-0.93	0.40	19.09	7.14E-05	2.32E-02
ENSG00000043514.17	TRIT1	1188.44	-0.92	0.24	16.86	2.19E-04	4.61E-02
ENSG00000137575.12	SDCBP	616.76	-0.91	0.26	23.94	6.33E-06	4.50E-03
ENSG00000166575.17	TMEM135	270.80	-0.90	0.41	22.32	1.42E-05	8.77E-03
ENSG00000197045.13	GMFB	465.59	-0.89	0.33	20.48	3.56E-05	1.62E-02
ENSG00000116489.13	CAPZA1	2492.82	-0.85	0.25	17.73	1.42E-04	3.57E-02
ENSG00000133835.16	HSD17B4	970.84	-0.85	0.21	18.95	7.68E-05	2.37E-02
ENSG00000135535.17	CD164	1101.65	-0.81	0.26	23.69	7.18E-06	4.77E-03
ENSG00000123505.17	AMD1	2613.63	-0.81	0.24	28.87	5.39E-07	8.78E-04
ENSG00000213639.10	PPP1CB	1483.96	-0.80	0.27	21.07	2.66E-05	1.40E-02
ENSG00000124333.16	VAMP7	1223.52	-0.80	0.25	18.22	1.11E-04	3.02E-02
ENSG00000177565.16	TBL1XR1	623.67	-0.79	0.40	16.88	2.16E-04	4.61E-02
ENSG00000163743.13	RCHY1	503.70	-0.79	0.30	20.45	3.63E-05	1.62E-02
ENSG00000111530.13	CAND1	1560.57	-0.71	0.30	16.79	2.26E-04	4.70E-02
ENSG00000084073.9	ZMPSTE24	1386.77	-0.68	0.25	21.20	2.49E-05	1.39E-02
ENSG00000261247.1	GOLGA8T	20.36	-0.67	1.18	16.61	2.47E-04	4.76E-02
ENSG00000119787.14	ATL2	636.20	-0.66	0.32	19.00	7.47E-05	2.35E-02
ENSG00000135968.20	GCC2	189.05	-0.61	0.46	22.39	1.37E-05	8.77E-03
ENSG00000275496.4	LOC102724701	48.52	0.09	0.82	42.37	6.31E-10	3.77E-06
ENSG00000130635.15	COL5A1	290.37	0.63	0.35	27.92	8.64E-07	1.19E-03
ENSG00000130940.15	CASZ1	581.09	0.74	0.23	36.03	1.50E-08	4.48E-05
ENSG00000155254.13	MARVELD1	500.88	0.80	0.36	19.07	7.24E-05	2.32E-02
ENSG00000135709.12	KIAA0513	269.09	0.82	0.41	18.27	1.08E-04	3.02E-02
ENSG00000102901.13	CENPT	1916.58	0.85	0.22	16.92	2.12E-04	4.61E-02
ENSG00000008710.19	PKD1	1529.40	0.95	0.23	17.28	1.77E-04	4.28E-02
ENSG00000151006.7	PRSS53	68.44	1.00	0.65	19.28	6.50E-05	2.30E-02
ENSG00000174705.13	SH3PXD2B	1138.35	1.08	0.27	40.15	1.91E-09	8.55E-06
ENSG00000277399.4	GPR179	20.53	1.16	1.65	16.69	2.37E-04	4.76E-02
ENSG00000187122.17	SLIT1	210.61	1.29	0.48	24.54	4.69E-06	4.00E-03
ENSG00000089154.11	GCN1	5266.75	1.47	0.29	26.36	1.89E-06	2.26E-03
ENSG00000196628.18	TCF4	240.24	2.21	0.56	24.03	6.04E-06	4.50E-03
ENSG00000167971.16	CASKIN1	636.11	2.33	0.44	27.43	1.11E-06	1.42E-03
ENSG00000160219.12	GAB3	64.96	2.35	0.67	20.10	4.32E-05	1.84E-02
ENSG00000285053.1	TBCE	155.22	4.23	0.82	24.59	4.58E-06	4.00E-03
ENSG00000198237.8	–	21.42	5.33	2.39	18.78	8.38E-05	2.54E-02
ENSG00000280987.4	MATR3	124.60	8.62	0.95	72.68	1.65E-16	1.48E-12
ENSG00000103426.12	CORO7-PAM16	65.29	10.84	1.62	79.71	4.90E-18	8.78E-14

Table S15. Overall differential gene expression after light-induction. indicating light vs non-light log2-fold changes shrunken using ashr[24]. (Fig. S18 displays values of Table S13 and S14 to show time-resolution).

ENSEMBL	Symbol	BaseMean	Log2FoldChange	lfcSE	pvalue	padj
ENSG00000280987.4	MATR3	124.60	-5.03	0.68	1.65E-16	1.48E-12
ENSG00000167971.16	CASKIN1	636.11	-0.15	0.44	1.11E-06	1.42E-03
ENSG00000135968.20	GCC2	189.05	-0.12	0.40	1.37E-05	8.77E-03
ENSG00000103426.12	CORO7-PAM16	65.29	-0.03	0.27	4.90E-18	8.78E-14
ENSG00000285053.1	TBCE	155.22	-0.01	0.17	4.58E-06	4.00E-03
ENSG00000188419.14	CHM	506.00	-0.01	0.09	3.41E-05	1.62E-02
ENSG00000023516.9	AKAP11	511.75	-0.01	0.09	2.92E-05	1.49E-02
ENSG00000261098.1	–	72.00	-0.00	0.08	3.36E-07	7.52E-04
ENSG00000078177.14	N4BP2	156.39	-0.00	0.05	2.08E-04	4.61E-02
ENSG00000123505.17	AMD1	2613.63	-0.00	0.02	5.39E-07	8.78E-04
ENSG00000162409.11	PRKAA2	1053.00	-0.00	0.03	7.14E-05	2.32E-02

ENSEMBL	Symbol	BaseMean	Log2FoldChange	lfcSE	pvalue	padj
ENSG00000196628.18	TCF4	240.24	-0.00	0.04	6.04E-06	4.50E-03
ENSG00000111530.13	CAND1	1560.57	-0.00	0.01	2.26E-04	4.70E-02
ENSG00000084073.9	ZMPSTE24	1386.77	-0.00	0.01	2.49E-05	1.39E-02
ENSG00000089154.11	GCN1	5266.75	-0.00	0.01	1.89E-06	2.26E-03
ENSG00000137575.12	SDCBP	616.76	-0.00	0.01	6.33E-06	4.50E-03
ENSG00000162601.11	MYSM1	1501.36	-0.00	0.02	7.21E-05	2.32E-02
ENSG00000177565.16	TBL1XR1	623.67	-0.00	0.02	2.16E-04	4.61E-02
ENSG00000213639.10	PPP1CB	1483.96	-0.00	0.01	2.66E-05	1.40E-02
ENSG00000143952.20	VPS54	374.66	-0.00	0.02	8.90E-05	2.61E-02
ENSG00000135535.17	CD164	1101.65	-0.00	0.01	7.18E-06	4.77E-03
ENSG00000116489.13	CAPZA1	2492.82	-0.00	0.01	1.42E-04	3.57E-02
ENSG00000134318.14	ROCK2	241.38	-0.00	0.02	1.11E-04	3.02E-02
ENSG00000115540.15	MOB4	559.47	-0.00	0.01	4.56E-06	4.00E-03
ENSG00000107290.14	SETX	611.62	-0.00	0.01	3.49E-05	1.62E-02
ENSG00000111860.14	CEP85L	359.31	-0.00	0.02	2.36E-04	4.76E-02
ENSG00000163743.13	RCHY1	503.70	-0.00	0.01	3.63E-05	1.62E-02
ENSG00000117569.18	PTBP2	1576.88	-0.00	0.01	4.37E-06	4.00E-03
ENSG00000124333.16	VAMP7	1223.52	-0.00	0.01	1.11E-04	3.02E-02
ENSG00000197045.13	GMFB	465.59	-0.00	0.01	3.56E-05	1.62E-02
ENSG00000198826.11	ARHGAP11A	301.12	-0.00	0.02	4.30E-07	7.70E-04
ENSG00000211456.12	SACM1L	347.14	-0.00	0.01	6.28E-05	2.30E-02
ENSG00000139618.15	BRCA2	389.39	-0.00	0.02	7.75E-07	1.16E-03
ENSG00000254535.4	PABPC4L	262.88	-0.00	0.01	1.07E-04	3.02E-02
ENSG00000137145.20	DENND4C	393.43	-0.00	0.02	1.28E-04	3.40E-02
ENSG00000134987.11	WDR36	707.44	-0.00	0.01	2.19E-04	4.61E-02
ENSG00000047410.14	TPR	1007.40	-0.00	0.01	4.06E-05	1.78E-02
ENSG00000198887.9	SMC5	554.70	-0.00	0.01	5.06E-05	1.97E-02
ENSG00000172795.16	DCP2	508.89	-0.00	0.01	6.82E-05	2.32E-02
ENSG00000139218.18	SCAF11	604.61	-0.00	0.01	2.69E-04	4.92E-02
ENSG00000153922.10	CHD1	649.21	-0.00	0.01	2.41E-04	4.76E-02
ENSG00000119787.14	ATL2	636.20	-0.00	0.01	7.47E-05	2.35E-02
ENSG00000153914.16	SREK1	694.96	-0.00	0.01	2.13E-04	4.61E-02
ENSG00000180008.9	SOCS4	390.43	-0.00	0.01	6.56E-05	2.30E-02
ENSG00000118496.5	FBXO30	279.56	-0.00	0.01	2.06E-04	4.61E-02
ENSG00000151006.7	PRSS53	68.44	-0.00	0.02	6.50E-05	2.30E-02
ENSG00000213186.8	TRIM59	259.19	-0.00	0.01	1.42E-04	3.57E-02
ENSG00000166575.17	TMEM135	270.80	-0.00	0.01	1.42E-05	8.77E-03
ENSG00000152942.19	RAD17	448.81	-0.00	0.01	5.87E-05	2.19E-02
ENSG00000117528.14	ABCD3	727.67	-0.00	0.01	2.66E-05	1.40E-02
ENSG00000137040.10	RANBP6	208.85	-0.00	0.01	2.45E-04	4.76E-02
ENSG00000144893.12	MED12L	301.03	-0.00	0.01	2.29E-04	4.72E-02
ENSG00000164023.14	SGMS2	201.14	-0.00	0.01	2.30E-05	1.37E-02
ENSG00000065615.14	CYB5R4	205.46	-0.00	0.01	2.69E-04	4.92E-02
ENSG00000160219.12	GAB3	64.96	-0.00	0.01	4.32E-05	1.84E-02
ENSG00000008710.19	PKD1	1529.40	-0.00	0.01	1.77E-04	4.28E-02
ENSG00000100554.12	ATP6V1D	456.45	-0.00	0.01	8.53E-05	2.55E-02
ENSG00000137573.14	SULF1	41.31	-0.00	0.01	5.87E-05	2.19E-02
ENSG00000186472.20	PCLO	219.03	-0.00	0.01	3.84E-07	7.64E-04
ENSG00000260317.1	–	56.91	-0.00	0.01	3.18E-06	3.56E-03
ENSG00000155097.12	ATP6V1C1	475.48	-0.00	0.01	1.60E-04	3.92E-02
ENSG00000188611.15	ASAH2	125.17	-0.00	0.01	1.31E-04	3.40E-02
ENSG00000151690.15	MFSD6	58.86	-0.00	0.01	4.59E-05	1.86E-02
ENSG00000229018.5	–	18.01	-0.00	0.01	1.30E-04	3.40E-02
ENSG00000043514.17	TRIT1	1188.44	-0.00	0.01	2.19E-04	4.61E-02
ENSG00000164294.14	GPX8	281.17	-0.00	0.01	5.58E-06	4.50E-03
ENSG00000261247.1	GOLGA8T	20.36	0.00	0.01	2.47E-04	4.76E-02
ENSG00000102901.13	CENPT	1916.58	0.00	0.01	2.12E-04	4.61E-02
ENSG00000139973.16	SYT16	34.02	0.00	0.01	3.78E-06	3.98E-03
ENSG00000198064.13	NPIPB13	21.84	0.00	0.01	6.53E-06	4.50E-03
ENSG00000275496.4	LOC102724701	48.52	0.00	0.01	6.31E-10	3.77E-06
ENSG00000144840.9	RABL3	210.33	0.00	0.01	2.05E-04	4.61E-02
ENSG00000198237.8	–	21.42	0.00	0.01	8.38E-05	2.54E-02
ENSG00000277399.4	GPR179	20.53	0.00	0.01	2.37E-04	4.76E-02
ENSG00000113558.18	SKP1	777.43	0.00	0.01	2.48E-05	1.39E-02
ENSG00000187122.17	SLIT1	210.61	0.00	0.01	4.69E-06	4.00E-03
ENSG00000133835.16	HSD17B4	970.84	0.00	0.01	7.68E-05	2.37E-02
ENSG00000118197.14	DDX59	146.59	0.00	0.01	2.63E-04	4.92E-02
ENSG00000165533.18	TTC8	412.62	0.00	0.01	2.64E-04	4.92E-02
ENSG00000126217.21	MCF2L	128.46	0.00	0.01	1.10E-04	3.02E-02
ENSG00000130635.15	COL5A1	290.37	0.00	0.01	8.64E-07	1.19E-03
ENSG00000155254.13	MARVELD1	500.88	0.00	0.01	7.24E-05	2.32E-02
ENSG00000104549.12	SQLE	741.09	0.00	0.01	3.01E-05	1.50E-02

ENSEMBL	Symbol	BaseMean	Log2FoldChange	lfcSE	pvalue	padj
ENSG00000135709.12	KIAA0513	269.09	0.00	0.01	1.08E-04	3.02E-02
ENSG00000136156.15	ITM2B	1743.03	0.00	0.01	4.47E-05	1.86E-02
ENSG00000119446.14	RBM18	796.72	0.00	0.01	2.95E-08	7.56E-05
ENSG00000196218.12	RYR1	44.14	0.00	0.02	2.03E-04	4.61E-02
ENSG00000174705.13	SH3PXD2B	1138.35	0.00	0.01	1.91E-09	8.55E-06
ENSG00000076043.10	REXO2	428.79	0.00	0.01	2.52E-04	4.80E-02
ENSG00000143499.14	SMYD2	292.08	0.00	0.01	1.98E-04	4.61E-02
ENSG00000283154.2	SCHIP1	201.62	0.00	0.01	4.67E-05	1.86E-02
ENSG00000109911.19	ELP4	554.11	0.00	0.01	2.43E-04	4.76E-02
ENSG00000130940.15	CASZ1	581.09	0.00	0.01	1.50E-08	4.48E-05
ENSG00000142178.9	SIK1	78.82	0.01	0.13	7.11E-05	2.32E-02
ENSG00000256591.5	–	73.85	0.01	0.11	1.44E-04	3.58E-02
ENSG00000259607.1	–	8.87	0.02	0.34	2.06E-04	4.61E-02
ENSG00000187664.9	HAPLN4	10.79	0.04	0.49	6.04E-06	4.50E-03
ENSG00000257225.1	–	14.13	0.04	0.49	5.55E-09	1.99E-05

SUPPLEMENTARY REFERENCES

[1] A. Munoz-Lopez, A. Jung, B. Buchmuller, J. Wolffgramm, S. Maurer, A. Witte, D. Summerer, *Chembiochem* **2020**, 22, 645.

[2] D. L. Bernstein, J. E. Le Lay, E. G. Ruano, K. H. Kaestner, *J. Clin. Invest.* **2015**, *125*, 1998.

[3] D. G. Gibson, L. Young, R.-Y. Chuang, J. C. Venter, C. A. Hutchison, H. O. Smith, *Nat. Methods* **2009**, *6*, 343.

[4] T. Cermak, E. L. Doyle, M. Christian, L. Wang, Y. Zhang, C. Schmidt, J. A. Baller, N. V. Somia, A. J. Bogdanove, D. F. Voytas, *Nucleic Acids Res.* **2011**, *39*, e82.

[5] E. A. Lemke, D. Summerer, B. H. Geierstanger, S. M. Brittain, P. G. Schultz, *Nat. Chem. Biol.* **2007**, *3*, 769.

[6] N. Wu, A. Deiters, T. A. Cropp, D. King, P. G. Schultz, *J. Am. Chem. Soc.* **2004**, *126*, 14306.

[7] R Core Team, *R: A Language and Environment for Statistical Computing*, **2019**, Vienna, Austria, can be found under http://www.R-project.org/.

[8] A. S. Matt Dowle, *data.table: Extension of data.frame. R package version 1.12.8*, **2019,** can be found under https://CRAN.R-project.org/package=data.table.

[9] a) H. Wickham, *ggplot2*, Springer International Publishing, Cham, **2016**; b) Baptiste Auguie, *gridExtra: Miscellaneous Functions for "Grid" Graphics*, **2017,** can be found under https://CRAN.R-project.org/package=gridExtra.

[10] L. Scrucca, M. Fop, T. B. Murphy, A. E. Raftery, *The R Journal* **2016**, *8*, 205.

[11] a) J. Schindelin, I. Arganda-Carreras, E. Frise, V. Kaynig, M. Longair, T. Pietzsch, S. Preibisch, C. Rueden, S. Saalfeld, B. Schmid et al., *Nat. Methods* **2012**, *9*, 676; b) C. A. Schneider, W. S. Rasband, K. W. Eliceiri, *Nat. Methods* **2012**, *9*, 671.

[12] A. Munoz-Lopez, B. Buchmuller, J. Wolffgramm, A. Jung, M. Hussong, J. Kanne, M. R. Schweiger, D. Summerer, *Angew. Chem. Int. Ed. Engl.* **2020**, 59, 8927.

[13] A. P. Masella, A. K. Bartram, J. M. Truszkowski, D. G. Brown, J. D. Neufeld, *BMC Bioinformatics* **2012**, *13*, 31.

[14] B. Bushnell, "BBMap: A Fast, Accurate, Splice-Aware Aligner", can be found under https://www.sourceforge.net/projects/bbmap/.

[15] M. Lawrence, W. Huber, H. Pagès, P. Aboyoun, M. Carlson, R. Gentleman, M. T. Morgan, V. J. Carey, *PLOS Comp. Biol.* **2013**, *9*, e1003118.

[16] T. Buschmann, L. V. Bystrykh, *BMC Bioinformatics* **2013**, *14*, 272.

[17] S. Palei, B. Buchmuller, J. Wolffgramm, Á. Muñoz-Lopez, S. Jung, P. Czodrowski, D. Summerer, *J. Am. Chem. Soc.* **2020**, *142*, 7289.

[18] A. Frankish, M. Diekhans, A.-M. Ferreira, R. Johnson, I. Jungreis, J. Loveland, J. M. Mudge, C. Sisu, J. Wright, J. Armstrong et al., *Nucleic Acids Res.* **2019**, *47*, D766-D773.

[19] R. Patro, G. Duggal, M. I. Love, R. A. Irizarry, C. Kingsford, *Nat. Methods* **2017**, *14*, 417.

[20] A. Srivastava, L. Malik, H. Sarkar, M. Zakeri, F. Almodaresi, C. Soneson, M. I. Love, C. Kingsford, R. Patro, *Alignment and mapping methodology influence transcript abundance estimation*, **2019**.

[21] A. Roberts, C. Trapnell, J. Donaghey, J. L. Rinn, L. Pachter, *Genome Biol.* **2011**, *12*, R22.

[22] a) M. I. Love, C. Soneson, P. F. Hickey, L. K. Johnson, N. T. Pierce, L. Shepherd, M. Morgan, R. Patro, *Tximeta: reference sequence checksums for provenance identification in RNA-seq*, **2019**; b) H. Pagès, M. Carlson, S. Falcon, N. Li, *AnnotationDbi: Manipulation of SQLite-based annotations in Bioconductor. R package version 1.48.0*, Bioconductor, **2019**.
[23] M. I. Love, W. Huber, S. Anders, *Genome Biol.* **2014**, *15*, 550.
[24] M. Stephens, *Biostatistics (Oxford, England)* **2017**, *18*, 275.
[25] M. Stephens, P. Carbonetto, D. Gerard, M. Lu, L. Sun, J. Willwerscheid, N. Xiao, *ashr: Methods for Adaptive Shrinkage, using Empirical Bayes. R package version 2.2-47.*, **2020,** can be found under https://cran.r-project.org/package=ashr.
[26] J. Cox, M. Mann, *Nat. Biotechnol.* **2008**, *26*, 1367.
[27] H. Pedersen, S. Hölder, D. P. Sutherlin, U. Schwitter, D. S. King, P. G. Schultz, *Proc. Natl. Acad. Sci. U. S. A.* **1998**, *95*, 10523.
[28] M. Rubino, P. Kunderfranco, G. Basso, C. M. Greco, F. Pasqualini, S. Serio, M. Roncalli, L. Laghi, A. Mantovani, R. Papait et al., *Oncoimmunology* **2017**, *6*, e1333215.

APPENDIX

Sequence of DNMT3a3L

NHDQEFDPPKVYPPVPAEKRKPIRVLSLFDGIATGLLVLKDLGIQVDRYIASEVCEDSITVGMV
RHQGKIMYVGDVRSVTQKHIQEWGPFDLVIGGSPCNDLSIVNPARKGLYEGTGRLFFEFYRLL
HDARPKEGDDRPFFWLFENVVAMGVSDKRDISRFLESNPVMIDAKEVSAAHRARYFWGNLP
GMNRPLASTVNDKLELQECLEHGRIAKFSKVRTITTRSNSIKQGKDQHFPVFMNEKEDILWCT
EMERVFGFPVHYTDVSNMSRLARQRLLGRSWSVPVIRHLFAPLKEYFACVSSGNSNANSRGP
SFSSGLVPLSLRGSHMGPMEIYKTVSAWKRQPVRVLSLFRNIDKVLKSLGFLESGSGSGGGTL
KYVEDVTNVVRRDVEKWGPFDLVYGSTQPLGSSCDRCPGWYMFQFHRILQYALPRQESQRP
FFWIFMDNLLLTEDDQETTTRFLQTEAVTLQDVRGRDYQNAMRVWSNIPGLKSKHAPLTPKE
EEYLQAQVRSRSKLDAPKVDLLVKNCLLPLREYFKYFSQNSLPL

human DNMT3A part (amino acids 612-912, with catalytic C710 in red)
S714, R736, E756, R771, T835, R836, R882 and W893 are highlighted in grey
Linker
mouse DNMT3L part (amino acids 208-421)

SatIII Sequence

TALE was assembled to recognize the antisense sequence highlighted in grey. Additional, the analyzed first CpG (of Fig. S14) and second CpG (of Fig. 3c) are shown.

Sense:

AATCAACCCGAGTGCAATCGAATGGAATCGAATGAATGGAATGCAATGGAATGGATTCA
ACTTGAATGGAATGGAAAGAATGGAATCAACACGAGTGGAATGGCATGGATTGGAATGG
AATGGAATGGAATCAACCCGAGTACAGGAATGGAATGGAA

Antisense:

TTCCATTCCATTCCTGTACTCGGGTTGATTCCATTCCATTCCATTCCAATCCATGCCATTCC
ACTCGTGTTGATTCCATTCTTTCCATTCCATTCAAGTTGAATCCATTCCATTGCATTCCATT
CATTCGATTCCATTCGATTGCACTCGGGTTGATT

Plasmid Maps

All plasmid maps were created with SnapGene (from Insightful Science; available at snapgene.com).

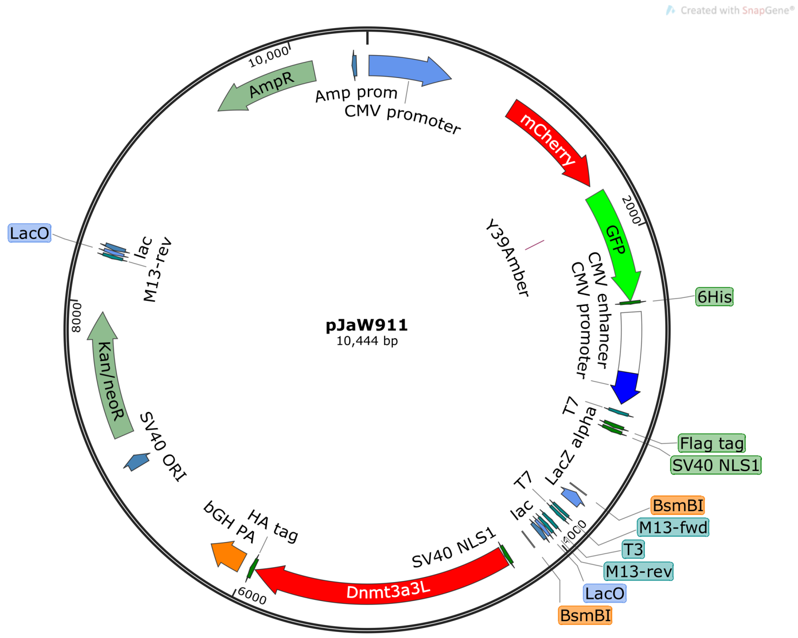

Figure S19. Plasmid map of pJaW911.

Golden Gate 2 entry plasmid with wild type DNMT3a3L.

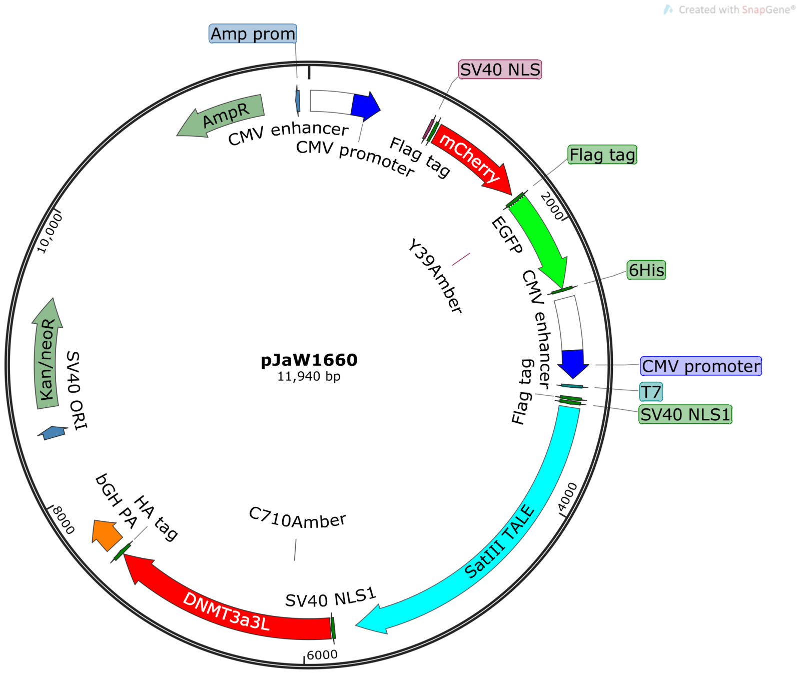

Figure S20. Plasmid map of pJaW1660.

SatIII-pcDNMT construct.

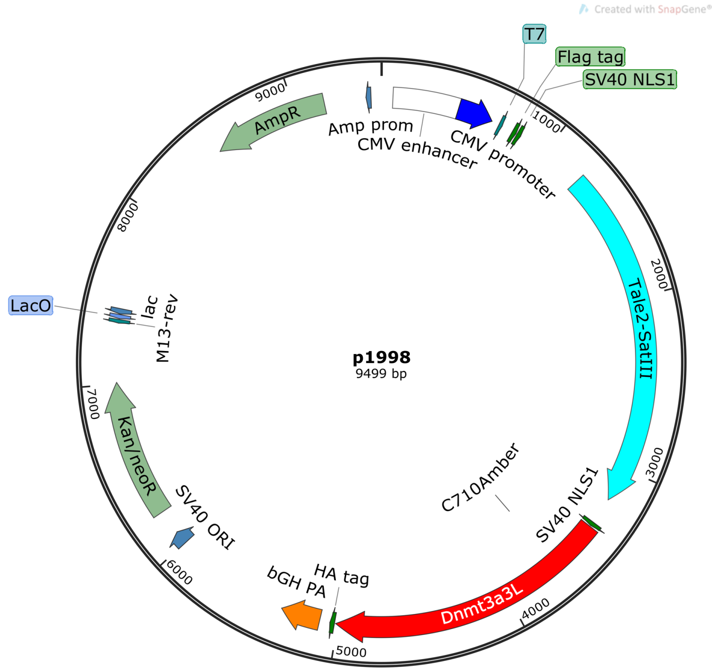

Figure S21. Plasmid map of pJaW1998.

SatIII-pcDNMT construct w/o transfection control.

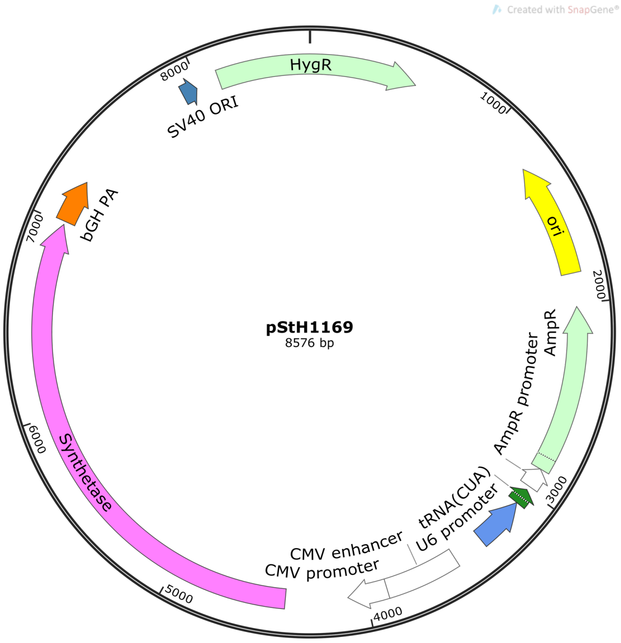

Figure S22. Plasmid map of pStH1169.

Encodes tRNA and synthetase pLRS_BH5.

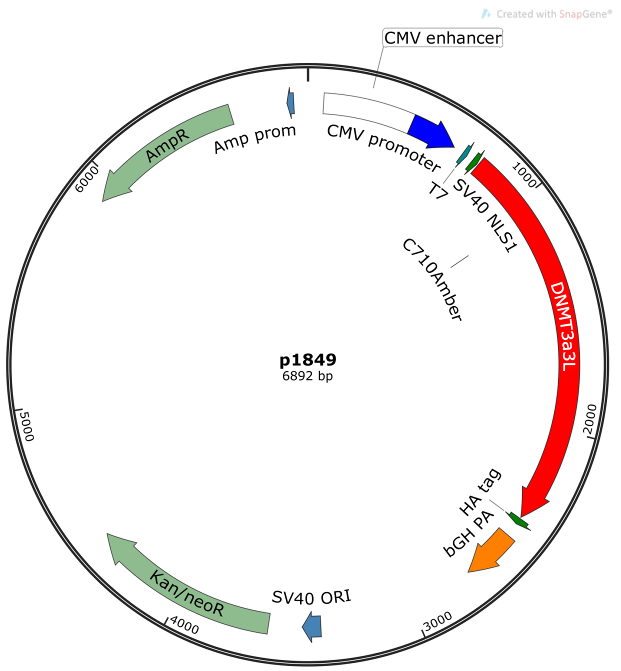

Figure S23. Plasmid map of pJaW1849.

Global pcDNMT3a3L construct w/o transfection control.
